# Enhanced Risk Stratification for Children and Young Adults with B-Cell Acute Lymphoblastic Leukemia: A Children’s Oncology Group Report

**DOI:** 10.1038/s41375-024-02166-1

**Published:** 2024-02-15

**Authors:** N. J. DelRocco, M. L. Loh, M. J. Borowitz, S. Gupta, K. R. Rabin, P. Zweidler-McKay, K. W. Maloney, L. A. Mattano, E. Larsen, A. Angiolillo, R. J. Schore, M. J. Burke, W. L. Salzer, B. L. Wood, A. J. Carroll, N. A. Heerema, S. C. Reshmi, J. M. Gastier-Foster, R. Harvey, I. M. Chen, K. G. Roberts, C. G. Mullighan, C. Willman, N. Winick, W. L. Carroll, R. E. Rau, D. T. Teachey, S. P. Hunger, E. A. Raetz, M. Devidas, J. A. Kairalla

**Affiliations:** 1 Department of Biostatistics, Colleges of Medicine, Public Health and Health Professions, University of Florida, Gainesville, FL USA; 2 Department of Population and Public Health Sciences, University of Southern California, Los Angeles, CA USA; 3 Department of Pediatrics and the Ben Towne Center for Childhood Cancer Research, Seattle Children’s Hospital, University of Washington, Seattle, WA USA; 4 Department of Pathology, Johns Hopkins University, Baltimore, MD USA; 5 Division of Haematology/Oncology, Hospital for Sick Children, University of Toronto, Toronto, ON Canada; 6 Division of Pediatric Hematology/Oncology, Texas Children’s Cancer Center, Baylor College of Medicine, Houston, TX USA; 7 Immunogen, Inc, Waltham, MA USA; 8 Department of Pediatrics, University of Colorado and Children’s Hospital Colorado, Aurora, CO USA; 9 HARP Pharma Consulting, Mystic, CT USA; 10 Department of Pediatrics, Maine Children’s Cancer Program, Scarborough, ME USA; 11 Servier Pharmaceuticals, Boston, MA USA; 12 Division of Pediatric Oncology, Children’s National Hospital, Washington, DC and the George Washington University School of Medicine and Health Sciences, Washington, DC, USA; 13 Division of Pediatric Hematology-Oncology, Medical College of Wisconsin, Milwaukee, WI USA; 14 Uniformed Services University, F. Edward Hebert School of Medicine, Bethesda, MD USA; 15 Children’s Hospital Los Angeles, Pathology and Laboratory Medicine, Los Angeles, CA USA; 16 Department of Genetics, University of Alabama at Birmingham, Birmingham, AL USA; 17 Department of Pathology, The Ohio State University Wexner School of Medicine, Columbus, OH USA; 18 Department of Pathology and Laboratory Medicine, Nationwide Children’s Hospital and Departments of Pathology and Pediatrics, Ohio State University College of Medicine, Columbus, OH USA; 19 Department of Pediatrics, Texas Children’s Cancer Center, Baylor College of Medicine, Houston, TX USA; 20 University of New Mexico Cancer Center, Albuquerque, NM USA; 21 Department of Pathology, St Jude Children’s Research Hospital, Memphis, TN USA; 22 Mayo Clinic, Cancer Center/Laboratory Medicine and Pathology, Rochester, NY USA; 23 UTSouthwestern, Simmons Cancer Center, Dallas, TX USA; 24 Perlmutter Cancer Center and Department of Pediatrics, NYU Langone Health, New York, NY USA; 25 Department of Pediatrics and The Center for Childhood Cancer Research, Children’s Hospital of Philadelphia and the Perelman School of Medicine at The University of Pennsylvania, Philadelphia, PA USA; 26 Department of Global Pediatric Medicine, St. Jude Children’s Research Hospital, Memphis, TN USA

**Keywords:** Cancer, Cancer

## Abstract

Current strategies to treat pediatric acute lymphoblastic leukemia rely on risk stratification algorithms using categorical data. We investigated whether using continuous variables assigned different weights would improve risk stratification. We developed and validated a multivariable Cox model for relapse-free survival (RFS) using information from 21199 patients. We constructed risk groups by identifying cutoffs of the COG Prognostic Index (PI_COG_) that maximized discrimination of the predictive model. Patients with higher PI_COG_ have higher predicted relapse risk. The PI_COG_ reliably discriminates patients with low vs. high relapse risk. For those with moderate relapse risk using current COG risk classification, the PI_COG_ identifies subgroups with varying 5-year RFS. Among current COG standard-risk average patients, PI_COG_ identifies low and intermediate risk groups with 96% and 90% RFS, respectively. Similarly, amongst current COG high-risk patients, PI_COG_ identifies four groups ranging from 96% to 66% RFS, providing additional discrimination for future treatment stratification. When coupled with traditional algorithms, the novel PI_COG_ can more accurately risk stratify patients, identifying groups with better outcomes who may benefit from less intensive therapy, and those who have high relapse risk needing innovative approaches for cure.

## Introduction

Outcomes among children with acute lymphoblastic leukemia (ALL) have steadily improved, and event-free and overall survival (OS) now exceed 85% and 90% [1]. Therapy for ALL is determined using established risk factors, balancing treatment intensity with prognosis to minimize overtreating patients with favorable risk, and undertreating patients with higher risk.

Informed risk group (RG) stratification is crucial for optimal therapy [2]. Contemporary risk stratification algorithms typically use threshold-defined dichotomous categories of clinically relevant risk factors including presenting white blood cell count (e.g., < vs. ≥50 × 10^9^/L; WBC) and minimal residual disease (e.g., < vs. ≥0.01%; MRD). The Children’s Oncology Group (COG) B-ALL algorithm includes National Cancer Institute (NCI) RG, clinical variables (extramedullary disease status and steroid pretreatment), sentinel favorable and unfavorable risk genetics (FRG and URG, respectively), flow cytometric MRD of peripheral blood on induction day 8 (D8 MRD), and marrow on induction day 29 (D29 MRD) and at end of consolidation (EOC) [3].

Assigning differing weights to individual risk factors or using continuous numerical rather than categorical values may more accurately predict relapse risk. The UKALL group used MRD as a continuous variable to develop a prognostic model that generated a continuous score (prognostic index_UKALL_, PI_UKALL_) predicting patient-level relapse risk [4, 5]. This model incorporated favorable and unfavorable genetics, and both presenting WBC and D29 MRD as continuous variables. An increase in the PI_UKALL_ score was strongly associated with relapse risk in a validation cohort of three European pediatric ALL trials (combined *n* = 2313) [5].

We conducted an external validation of the PI_UKALL_ in >20000 COG trial participants and subsequently assessed the value of D8 MRD added to this model given our prior work showing the prognostic value of D8 MRD in certain patient subsets [6]. In contrast to the UK group, COG conducts different B- and T-ALL trials [7]. Thus, we focused on B-ALL and developed a novel risk score, PI_COG_, and compared patient outcomes between current and PI_COG_-derived RGs.

## Methods

### Study population

**Fig. 1:**
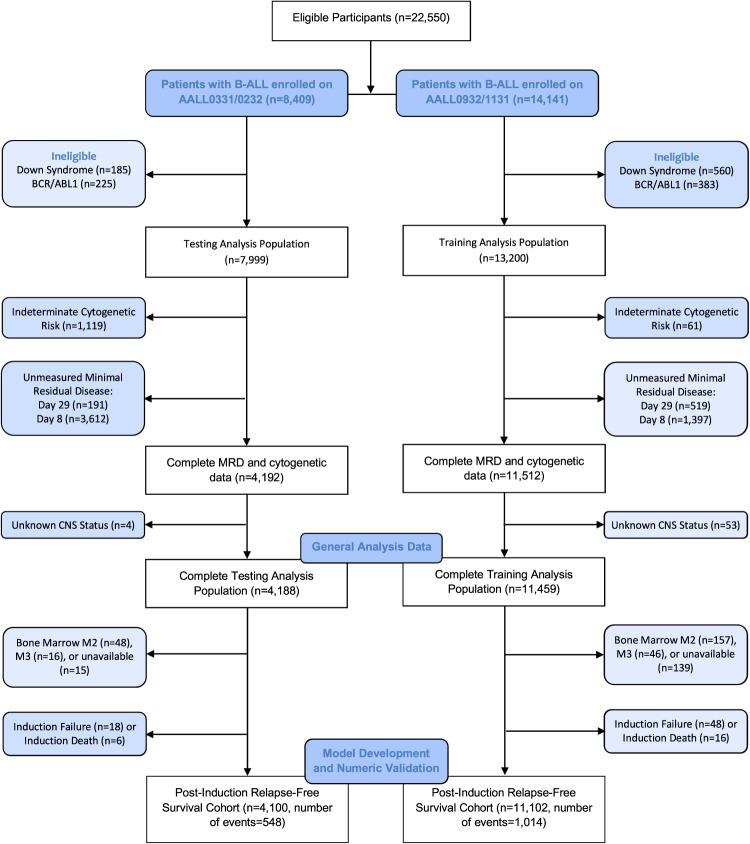
CONSORT diagram for testing (AALL0331/0232) and training (AALL0932/1131) therapeutic trials. B-ALL, B-Cell Acute Lymphoblastic Leukemia; BCR/ABL1, Philadelphia chromosome-positive ALL by BCR-ABL1 oncoprotein; MRD, minimal residual disease; Day 29, end-of-induction minimal residual disease; Day 8, induction day 8 minimal residual disease; CNS, central nervous system involvement; Bone marrow M1, <5% lymphoblasts (remission) Bone marrow M2, 5–25% lymphoblasts; Bone marrow M3, >25% lymphoblasts. Of note, day 8 PB MRD testing was not routinely measured for earlier trials (AALL0331/0232) until partway through accrual.

The cohort included 13,875 NCI standard-risk (SR) and 7324 NCI high-risk (HR) non-infant B-ALL patients enrolled on four COG trials from 2004-2019; two for SR and two for HR patients: AALL0331 (SR; *n* = 5099) [8], AALL0232 (HR; *n* = 2900) [9], AALL0932 (SR; *n* = 8776) [10], and AALL1131 (HR; *n* = 4424) [3, 11]. Patients and/or their caregiver(s) provided informed consent for these trials in accordance with the NIH central IRB and the Declaration of Helsinki. Randomizations differed for each trial. In all trials except AALL0232, primary analyses indicated no statistical differences in disease-free survival (DFS) rates between experimental treatment and standard of care arms [3, 8–11]. Down syndrome and Philadelphia chromosome-positive (Ph+) patients were excluded. Patients with T-ALL will be considered separately in future work. The CONSORT diagram shows the breakdown of study participants in each group and the final analysis population (Fig. 1).

### Variable selection methods

Choice of predictor variables is a crucial step when building a clinical prediction model. Investigators typically must reduce a larger set of candidates to a final set of predictor variables used for final model estimation. The methods of predictor selection can be classified into two categories: (1) reduction before modeling and (2) reduction while modeling [12]. Method (1) implies that the predictors are selected based on domain expertise prior to studying the relationship between the outcome and candidate predictors in the data to be used for model building. This method of predictor selection is generally preferred, as it best preserves the statistical properties of later model estimation and hypothesis testing [13]. Method (2) implies that knowledge of the relationship between the outcome and candidate variables in the data is used to select predictors. Examples of method (2) include univariable screening and stepwise selection (forward, backward, and combined). Though occasionally justifiable, the disadvantages of stepwise selection are well documented and include unstable selection; misleading bias in regression coefficients, standard errors, and p-values; and poorer predictions relative to a full model [12, 13]. Univariable screening inherits the same disadvantages as forward stepwise selection but tends to have poorer performance due to neglect of marginally “insignificant” variables [12]. Therefore, in this work, predictor variables (described in detail below) were selected for inclusion in the model a priori based on clinical expertise.

### Potential predictive variables

Known prognostically important genetic variables including *ETV6::RUNX1* fusions, double trisomies of chromosome 4, 10 (DT), intrachromosomal amplification of chromosome 21 (iAMP21), and *KMT2A*-rearrangements were determined by fluorescence in situ hybridization. Hypodiploid ALL was defined as modal chromosome number <44 or DNA index <0.81. These genetic variables determined the FRG and URG groups (detailed in Supplementary Methods). D8 and D29 MRD were measured by flow cytometry, as described [6]. CNS status was treated as a categorical variable: CNS1 (no blasts), CNS2 (CSF WBC < 5/µL with blasts), or CNS3 (CSF WBC ≥ 5/µL with blasts) (Supplementary Methods).

Transformations for WBC (log(WBC); WBC_log_), D8 MRD, and D29 MRD were consistent with Enshaei et al. [5] due to reasonable performance in the PI_UKALL_ model and clinical knowledge regarding their distributions. Transformed MRD is displayed as τ(MRD), corresponding roughly to the negative log transformation [5]. The maximum τ(MRD) was 13.82, corresponding to MRD < 1.0 × 10^−5^. Candidate predictor variables are shown in Supplementary Table 1.

### External validation of PI_UKALL_

Steps for external validation followed published guidelines [14]. These steps and the level of information required for each step’s execution are defined in Supplementary Table 2 and are referred to as Step(1)-Step(6). Note Step(5) and Step(6) are not included due to unavailable information. If in Step(1) the overall calibration slope is found to be less than one, the model is technically considered not to be optimal for the external validation data, though further steps should still be examined as the model may still have practical utility.

We applied the published PI_UKALL_ equation to the COG data [5]:

$${{{{{{\rm{PI}}}}}}}_{{{{{{\rm{UKALL}}}}}}}=	 \, [-0.218 \, {^{\ast}} \, {{{{{\rm{\tau }}}}}}\left({{{{{\rm{D}}}}}}29{{{{{\rm{MRD}}}}}}\right)-0.440 \, {^{\ast}} \, {{{{{\rm{CYTO}}}}}}\_{{{{{\rm{GR}}}}}}\\ 	+1.066 \, {^{\ast}} \, {{{{{\rm{CYTO}}}}}}\_{{{{{\rm{HR}}}}}} +0.138 \, {^{\ast}} \, {{{{{\rm{WBC}}}}}}_{\log }]$$

According to Step(1), the overall calibration slope for the PI_UKALL_ was calculated. The calibration slope is the estimated log-hazard ratio from a univariable Cox model with the PI_UKALL_ as the predictor. A calibration slope less than 1 in external validation data is indicative of poorer discrimination in the validation data than in the development data, a common occurrence among predictive models reflecting decreased generalizability of the original model and heterogeneity of patient prognosis in derivation vs. validation populations [14]. A formal test for the null hypothesis that the overall calibration slope equaled one was conducted.

For Step(2), the primary metric used to compare model discrimination was the concordance index (C-index), defined as the proportion of randomly selected pairs of patients that the model orders concordantly (for a pair to be concordant, the patient with the higher model-predicted probability of relapse has the shorter observed time to relapse) [15]. A C-index >0.7 indicates acceptable discriminative capability of a model, while a value of 0.5 indicates that prediction is equivalent to random chance [13]. In Step(3), ideally, the original published coefficients would be equal to those obtained if the model were refit in the external validation data. We examined the coefficients for the PI_UKALL_ model re-derived in the full COG analysis population compared to the published coefficients to examine possible true difference in predictor effects between UKALL and COG data. We conducted a hypothesis test for equality of published vs. externally derived model coefficients as detailed in Supplementary Table 2. Kaplan-Meier curves within PI_UKALL_-defined RGs were reported to satisfy Step(4).

### Added value of D8 MRD

D8 MRD is of particular interest to COG, as its collection is unique and standard within the collective. Therefore, to assess incremental added predictive value of D8 MRD to the UKALL model, we fit a multivariable Cox proportional hazards model including τ(D29 MRD), FRG, URG, WBC_log_ and compared this to the model with τ(D8 MRD) included.

### Development of PI_COG_

The development of a new prognostic index for relapse risk, the PI_COG_, utilized pre-specified covariates based on domain expertise and existing literature from UKALL and COG data [6]. The AALL0932/AALL1131 cohort comprised training data, while AALL0331/AALL0232 patients were used as testing data for temporal (external) validation (Fig. 1). For model development on the training data, τ(D29 MRD), FRG, URG, WBC_log_, τ(D8 MRD), age at diagnosis (Age), and CNS status were included from an initial class of potential covariates (Supplementary Table 1) due to existing evidence of prognostic relevance and current risk stratification algorithms.

Graphical methods assessed the assumptions of the functional relationships between relapse risk and covariates [13]. The proportional hazards assumption was examined using scaled Schoenfeld residual plots by covariate. Plots of the delta-beta residuals helped to visually identify participants with strong influence on hazard ratio estimation. We pre-specified a comprehensive set of potential interactions among the continuous variables (Supplementary Table 1) and assessed them for possible model inclusion as a group. PI_COG_ was defined as the linear predictor from the model. Calibration slopes and C-indices were obtained for PI_COG_ overall and within sex and race/ethnicity groups to diagnose potential lack of model fit.

Validation and calibration were assessed using the *rms* package in R [16]. The final model was internally validated using bootstrapping with B = 1000 resamples with optimism-corrected estimates calculated [15]. Calibration was examined using smoothed calibration plots [13]. Cox model performance was compared to machine learning (ML) alternatives to assess whether relaxed assumptions improved predictive ability. Random forest [17], support vector machine [18], and boosted Cox models were fit to the same predictor variables included in the Cox model (Supplementary Table 3) [19]. The benchmarking study included a 5×5-fold nested cross-validation routine adapted from Fouodo et al. [18].

To compare possible risk stratification approaches, patients were classified according to the current risk classification algorithms used in COG AALL1731 (SR; NCT03914625) and AALL1732 (HR; NCT03959085) trials (Supplementary Table 4, 5). Using the training dataset, cutpoints were calculated dividing the continuous PI_COG_ into four risk-based categories optimizing the model’s discriminative ability [20]. The censored nature of the data was accounted for by maximizing the Concordance Probability Estimate (CPE), a variation of the C-index [20]. Further details of how cutpoints were calculated are included in the Supplementary Methods. Point estimates of 5-year relapse-free survival (RFS) within risk subgroups were obtained using Kaplan-Meier estimation. RFS was defined as time from end of induction (EOI) to relapse or death in remission, or censored at second malignant neoplasm (SMN) or date of last contact for those who remained event-free. Estimates for DFS and OS were also obtained. DFS was defined as time from EOI to relapse, death in remission, or SMN, or censored at last contact. OS was defined as the time from EOI to death or censored at last contact. All analyses were conducted using R Statistical Software® version 4.2.1 (code available from corresponding author upon request) [21].

## Results

### Study population

**Fig. 2:**
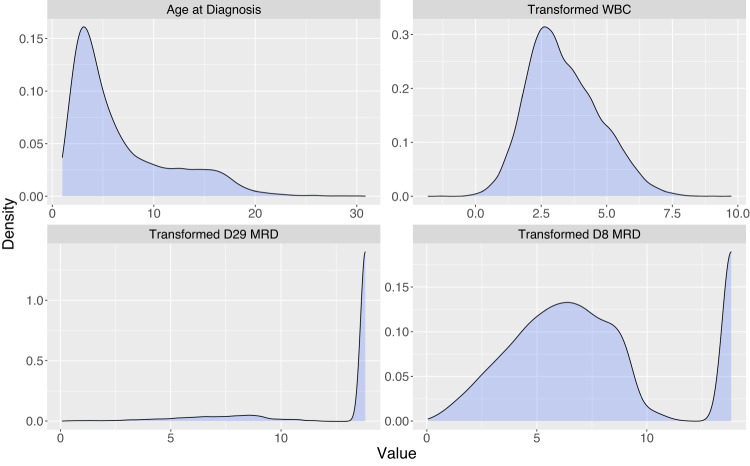
Density plots of the distribution of continuous prognostic variables for the full analysis population (*n* = 21199). White blood cell count (WBC) was log-transformed. End-of-induction (D29) minimal residual disease (MRD) and induction day 8 (D8) MRD were transformed according to UKALL [4, 5].

Overall, the distributions of clinical characteristics were similar between the training and testing data in both the generating analysis population (Table 1) and between the training and testing data in the post-induction relapse-free survival cohort used for model development and numeric validation (Supplementary Table 6). Among genetic groups, 9629 participants (45.4%) were FRG (52.1% *ETV6::RUNX1* fusions and 48.2% DT) and 1256 participants (5.9%) were URG (29.0% *KTM2A*-rearranged, 28.0% hypodiploid, and 43.4% iAMP21). Ph-like ALL (Supplementary Methods) was present in 996 of 4836 patients tested. D8 MRD and D29 MRD data were available for 76.4% and 84.4% of patients, respectively. Figure 2 shows the distribution of continuous prognostic factors for the combined population.

**Table 1:** Patient characteristics of the analysis population (*n* = 21199)^a^.

	Testing (*n* = 7999)	Training (*n* = 13,200)	Total (*n* = 21,199)
Age in years, median (range)	4.83 (1.0, 30.8)	4.83 (1.0, 30.8)	4.83 (1.0, 30.8)
Sex (%)			
Female	3666 (45.8)	5994 (45.4)	9660 (45.6)
Male	4333 (54.1)	7206 (54.6)	11539 (54.4)
NCI Risk (%)			
SR	5153 (64.4)	8959 (67.9)	14112 (66.6)
HR	2841 (35.5)	4241 (32.1)	7082 (33.4)
WBC x 1000/µl, median (range)	9.00 (0.06, 1306.0)	8.60 (0.1, 6200.0)	8.70 (0.06, 6200.0)
CNS (%)			
CNS1	7058 (88.2)	11580 (87.7)	18638 (87.9)
CNS2	816 (10.2)	1396 (10.6)	2212 (10.4)
CNS3	115 (1.4)	159 (1.2)	274 (1.3)
Race (self-declared) (%)			
Asian	355 (4.4)	607 (4.6)	962 (4.5)
Black	512 (6.4)	732 (5.6)	1244 (5.9)
White	6016 (75.2)	9630 (73.0)	15646 (73.8)
Other	78 (1.0)	292 (2.2)	370 (1.8)
Ethnicity (self-declared) (%)			
Hispanic	1730 (21.6)	3344 (25.3)	5074 (23.9)
Non-Hispanic	5932 (74.2)	9201 (69.7)	15133 (71.4)
Unknown	337 (4.2)	655 (5.0)	992 (4.7)
Cytogenetics (%)			
* ETV6::RUNX1*	1961 (24.5)	3372 (25.6)	5333 (25.2)
Double Trisomy	1807 (22.6)	3038 (23.0)	4845 (22.9)
* iAMP21*	165 (2.1)	380 (2.9)	545 (2.6)
Hypodiploidy	134 (1.7)	218 (1.7)	352 (1.7)
Ph-like^b^	239	757	996
* KMT2Ar*	147 (1.8)	217 (1.6)	364 (1.7)
PB MRD Day 8 (%)			
<0.01%	829 (10.4)	2716 (20.6)	3545 (16.7)
0.01-<0.1%	1104 (13.8)	3136 (23.8)	4240 (20.0)
0.1 to <1.0%	1338 (16.7)	3424 (25.9)	4762 (22.5)
>/= 1.0%	1116 (14.0)	2527 (19.1)	3643 (17.2)
Unknown	3612 (45.2)	1397 (10.6)	5009 (23.6)
BM MRD Day 29 (%)			
<0.01%	6078 (76.0)	9937 (75.3)	16015 (75.6)
0.01-<0.1%	824 (10.3)	1322 (10.0)	2146 (10.1)
0.1 to <1.0%	581 (7.3)	876 (6.6)	1457 (6.9)
>/= 1.0%	325 (4.1)	546 (4.1)	871 (4.1)
Unknown	191 (2.4)	519 (3.9)	710 (3.4)
Event type (%)			
None	6740 (84.3)	11,678 (88.5)	18,418 (86.9)
Induction Death	72 (0.9)	102 (0.8)	174 (0.8)
Induction Failure	35 (0.4)	71 (0.5)	106 (0.5)
Relapse	928 (11.6)	1068 (8.1)	1996 (9.4)
Remission Death	154 (1.9)	220 (1.7)	374 (1.8)
Second Malignant Neoplasm	70 (0.9)	61 (0.5)	131 (0.6)

^a^Ph+ and Down Syndrome patients excluded; Abbreviations: MRD, minimal residual disease; Race “Other” includes: Native Hawaiian/other Pacific Islander, American Indian or Alaska Native, and Multiple Races.

^b^Ph-Like testing was not conducted uniformly on all patients, therefore percentages are omitted as they may not indicate a representative proportion.

### External validation of PI_UKALL_

The calibration slope for the original PI_UKALL_ applied to COG data using the published coefficients was 0.79, which was significantly different from one (*p* < 0.001). The original PI_UKALL_ retained discrimination ability, with a C-index of *C* = 0.725. When the coefficients for the PI_UKALL_ model were recalculated using external COG data, FRG and WBC have larger hazard ratio estimates and D29 MRD and URG have smaller estimates, indicating possible differing predictor variable effect weighting between the two populations [derived PI_UKALL_ = −0.136*τ(D29 MRD)-0.913*FRG + 0.692*URG + 0.166*WBC_log_], and the risk directions for all factors were consistent between the original and derived PI_UKALL_. For example, FRG is associated with lower relapse risk in both cohorts. These coefficients (log-hazard ratios) associated with the derived PI_UKALL_ yield the following hazard ratios: 0.87 for τ(D29 MRD), 0.40 for FRG, 1.99 for URG, and 1.18 for WBC_log_. The test for equality of published vs. externally derived model coefficients showed evidence of difference in the coefficients (*p* < 0.001), indicating that model fit could be improved. Kaplan-Meier curves within PI_UKALL_-defined RGs are shown in Supplementary Fig. 1 and exhibit good separation between curves (log-rank *p* < 0.001).

### Added value of D8 MRD

τ(D8 MRD) was a statistically significant addition to the model, with a modest hazard ratio estimate in the testing data of 0.96 (1 DF Wald *p* < 0.001) (Supplementary Table 7). The effect size corresponds to an estimated 4% relapse risk reduction for a one-unit increase in τ(D8 MRD) (decrease in D8 MRD), holding D29 MRD, WBC_log_, FRG, and URG constant.

### Development of PI_COG_

We next developed a model using COG predictors best known for relapse risk using the training dataset (*n* = 11,102). Tested as a group, the set of potential statistical interactions did not significantly improve model fit (Supplementary Table 1) and were excluded. Table 2 reports the estimated coefficients and hazard ratios from the model containing transformed D8 and D29 MRD, FRG, URG, WBC_log_, CNS status, and Age. Except for CNS3 (*n* = 90 in training data, Supplementary Table 6) vs. CNS1, each predictor was strongly associated with relapse risk. Increases in transformed D8 and D29 MRD (i.e., decreases in MRD) were each associated with a decreased relapse risk. Table 2 can also be visualized as an equation as follows:

$${{{{{{\rm{PI}}}}}}}_{{{{{{\rm{COG}}}}}}}=	 \, [\!-0.102 \, {^{\ast}} \, {{{{{\rm{\tau }}}}}}\left({{{{{\rm{D}}}}}}29{{{{{\rm{MRD}}}}}}\right)-0.040 \, {^{\ast}} \, {{{{{\rm{\tau }}}}}}\left({{{{{\rm{D}}}}}}8{{{{{\rm{MRD}}}}}}\right)\!-0.741 \, {^{\ast}} \, {{{{{\rm{FRG}}}}}}\\ 	+0.644 \, {^{\ast}} \, {{{{{\rm{URG}}}}}}+0.156 \, {^{\ast}} \, {{{{{{\rm{WBC}}}}}}}_{\log }+0.386 \, {^{\ast}} \, {{{{{\rm{I}}}}}}({{{{{\rm{CNS}}}}}}2)\\ 	+0.364 \, {^{\ast}} \, {{{{{\rm{I}}}}}}({{{{{\rm{CNS}}}}}}3)+0.061 \, {^{\ast}} \, {{{{{\rm{Age}}}}}}]$$

where indicator I(CNS Status) is one if the patient falls into that CNS category, and zero otherwise. This equation can be used to calculate an individual patient’s PI_COG_ risk score. Supplementary Fig. 2 provides a visual comparison of the shapes of the distributions of PI_UKALL_ and PI_COG_. Figure 3 portrays the prognostic index by genetic RG, with higher genetic risk associated with higher PI_COG_.

**Table 2:** Summaries of the PI_COG_ model derived on the training study population.

Variable	Type	Coefficient	HR (95% CI)	*P*-value
τ(D29 ΜRD)	Continuous	−0.102	0.90 (0.89–0.92)	<0.001
τ(D8 ΜRD)	Continuous	−0.040	0.96 (0.94–0.98)	<0.001
FRG	Binary	−0.741	0.48 (0.41–0.56)	<0.001
URG	Binary	0.644	1.91 (1.59–2.29)	<0.001
WBC_log_	Continuous	0.156	1.17 (1.12–1.22)	<0.001
CNS Status	Categorical			
(Ref = CNS1)	CNS2	0.386	1.47 (1.24–1.74)	<0.001
	CNS3	0.364	1.44 (0.88–2.37)	0.151
Age Dx	Continuous	0.061	1.06 (1.05–1.07)	<0.001

**Fig. 3:**
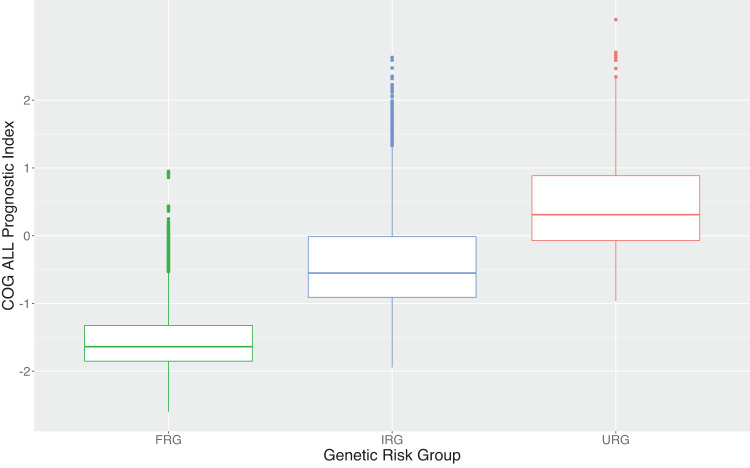
Boxplots of the distribution of the COG ALL Prognostic Index (PI_COG_) risk score by genetic risk group. The central “box” is made up of the 25th percentile, median (50th percentile), and 75th percentile. Lines on either side extend to the minimum and maximum (excluding outliers). Outliers are marked on the plot by points that are higher than the maximum denoted by the upper line.

Diagnostic plots indicate no concerning evidence of non-proportional hazards (Supplementary Fig. 3A) or influential points (Supplementary Fig. 3B). Internal validation indicated very little data-driven overfitting in the modeling process (Supplementary Table 8). Temporal external validation of the new model in the AALL0232/AALL0331 testing data (*n* = 4100) yielded an overall calibration slope of 0.94, not significantly different from 1 (*p* = 0.13), indicating overall good calibration in the testing dataset. The model held discrimination as well, with a C-index in the testing data of 0.738. Calibration curves are displayed in Supplementary Fig. 4. In testing data stratified by protocol, we observed a slight underestimation of risk among the few NCI HR patients (AALL0232) with very poor observed risk, likely due to the lack of sufficient data to obtain reliable predictions. Among NCI SR patients (AALL0331), there was an overestimation of risk across the range of the data, with the poorest model estimates again in ranges with fewer observations. The final Cox model was compared to ML alternatives using the same prognostic variables (Supplementary Table 3). Despite enhanced flexibility in the ML models, the discriminative ability of the Cox model was comparable to all ML alternatives.

### Comparison of risk stratification for PI_COG_ vs. COG current clinical

**Fig. 4:**
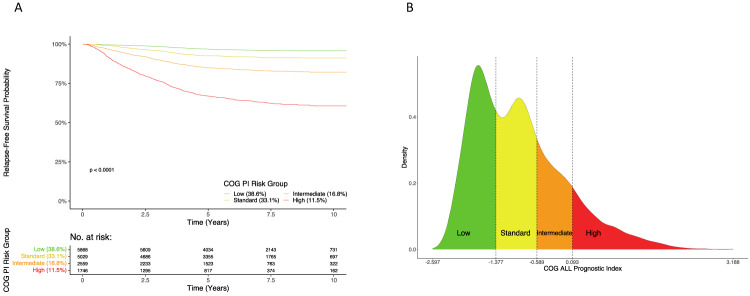
Summaries of the concordance probability estimator (CPE)-defined risk groups of the PI_COG_. **A** Kaplan-Meier Curves for Relapse-Free Survival probability within each PI_COG_-defined risk group for the combined RFS cohorts (*n* = 15202) and corresponding risk table. **B** Density plots of the distribution of the PI_COG_ with CPE-defined risk groups indicated by text (Low, Standard, Intermediate, and High) and color for the combined relapse-free survival (RFS) cohort (*n* = 15202). Risk group defining cutpoints of the PI_COG_ that maximize the CPE are marked by dashed vertical lines.

The cutpoints maximizing the CPE for PI_COG_ were −1.377, −0.589, and 0.093, resulting in classification of patients’ relapse risk into: 38.6% of patients as “low” (RFS 96.8%); 33.1% “standard” (92.6%); 16.8% “intermediate” (84.9%); and 11.5% “high” (66.9%). Figure 4A shows excellent separation and sensible RFS estimates among Kaplan-Meier curves within PI_COG_-defined RGs. Supplementary Fig. 5 displays the Kaplan-Meier curves within PI_COG_-defined RGs stratified by testing and training datasets, showing well-separated curves within each dataset. These stratified Kaplan-Meier curves are overlaid for comparison in Supplementary Fig. 6. Figure 4B demonstrates the practical implications of splitting patients’ prognostic values by RG, with each patient’s PI_COG_ value falling into one of the four risk categories depending on prognostic features. The distribution of the PI_COG_ is similar when stratified by testing and training datasets (Supplementary Fig. 7).

Ninety-seven percent of patients had sufficient data to be retrospectively classified according to current COG AALL1731/AALL1732 definitions. Shown in Supplementary Table 9, the resulting classification gives 24.5% SR-Favorable (5-year RFS 96.7%), 20.5% SR-Average (93.3%), 12.5% SR-High (82.7%), 3.0% HR-Favorable (96.3%), 29.6% HR (81.8%), and 1.1% Very HR (VHR; 53.6%). Table 3 compares the classification of patients according to both the PI_COG_ and the COG current clinical standard. As seen in the SR-Fav and VHR rows, the two risk classification strategies generally agree when risk is very high or very low. However, for other current COG risk classifications (SR-Avg, SR-High, HR) that collectively include 63% of patients, there is a broader spectrum of PI_COG_ RG assignment.

**Table 3:** Sample sizes (%) for subgroups by COG risk and COG Prognostic Index classification in the combined training/testing data.

	COG PI Classification				
COG Risk Classification	Low (5 Yr. RFS = 96.83%)	Standard (92.55%)	Intermediate (84.94%)	High (66.87%)	Total
SR Fav	4746 (93.48%)	331 (6.52%)	0 (0.00%)	0 (0.00%)	5077
SR-Avg	632 (16.73%)	2863 (75.80%)	282 (7.47%)	0 (0.00%)	3777
SR High	28 (1.31%)	669 (31.33%)	989 (46.32%)	449 (21.03%)	2135
HR Fav	219 (55.44%)	176 (44.56%)	0 (0.00%)	0 (0.00%)	395
HR	243 (6.62%)	990 (26.96%)	1287 (35.05%)	1152 (31.37%)	3672
VHR	0 (0.00%)	0 (0.00%)	0 (0.00%)	145 (100.00%)	145
Total	5868	5029	2558	1746	15,201

Table 4 displays 5-year RFS estimates within each of the subgroups discussed above. Within the COG SR-Avg group, PI_COG_ identified a “low risk” subgroup with an outstanding 96.0% RFS estimate, similar to the outcomes for patients traditionally classified as SR-Fav. In the COG HR group, we observed a broad range of RFS estimates, from a group with an RFS of 95.5% to a group with an RFS similar to that expected with VHR (66.0% RFS). Similar trends are seen for DFS and OS, as well as when the results are stratified by testing and training datasets (Supplementary Tables 10–19).

**Table 4:** 5-year relapse-free survival probability estimates (SE) for subgroups by COG retrospective and COG Prognostic Index risk classifications in the combined training/testing data.

	COG PI Classification			
COG Risk Classification	Low	Standard	Intermediate	High
SR Fav	0.970 (0.003)	0.927 (0.015)	--	--
SR Avg	0.960 (0.008)	0.937 (0.005)	0.904 (0.018)	--
SR High	0.924 (0.052)^a^	0.899 (0.012)	0.831 (0.013)	0.732 (0.022)
HR Fav	0.982 (0.009)	0.964 (0.014)	--	--
HR	0.955 (0.014)	0.903 (0.010)	0.850 (0.010)	0.660 (0.015)
VHR	--	--	--	0.534 (0.044)

Empty cells indicate insufficient sample size for reliable estimation (<25 patients).

Patients in SR-Fav/Avg are missing MRD8, as such they are not represented in this table.

^a^Large standard error reflects small sample size (*n* = 28) and hence broader uncertainty about the RFS estimate.

## Discussion

Prognostic models are used in oncology to aid clinical decision making by adjusting treatment intensity to individual patient relapse risk [22]. A prognostic model must satisfy many quality control guidelines to be useful in clinical practice, including appropriate model validation [12, 13, 15]. Ideally, this includes both strong resampling-based internal validation (“training”) and external validation in independent populations (“testing”) [12, 15].

We have developed and rigorously validated a new model to determine a prognostic index (PI_COG_) using COG B-ALL trials. PI_COG_ is easily calculated on a large scale and can be hosted online on a web-application for use by patients and practitioners, lending itself well to the described clinical applications (see https://natalie-delrocco.shinyapps.io/COG_PI_Calculator/). This work extends that of Enshaei et al., whose prognostic index, the PI_UKALL,_ was prognostic in the COG data and emphasized the strength of D29 MRD, WBC, and favorable and unfavorable cytogenetics as predictors of outcome in pediatric ALL [5].

These independent analyses were both conducted with large, uniformly annotated clinical trial datasets, giving strong evidence of reliable estimation of the effect of these prognostic factors on relapse risk. This work provided an independent external validation of the PI_UKALL,_ and also demonstrated the contributions of Age, CNS status, and D8 MRD in prognostic modeling for relapse risk. Despite correlation with D29 MRD and a modest effect, D8 MRD still contributes independently to the model, likely due to ability to indicate excellent expected outcomes when D8 MRD is negative. We additionally note that model estimation showing similar hazard ratios for patients with CNS2 and CNS3 is not unique to this study, and refer the interested reader to Winick et al. for discussion [23]. However, present interpretations regarding CNS2 vs. CNS3 must be made with caution as the confidence interval associated with the estimated hazard ratio for CNS3 patients (vs. CNS1) is wide given the relatively small number of these patients.

Difference in performance of PI_UKALL_ in COG patient populations may be attributed to several factors including different geographic case-mix [14], different MRD detection methods, and differing definitions of genetic factors [24]. Differences in cytogenetic classification between the COG and UKALL groups include the definition of hyperdiploidy. While the UKALL group defines this favorable cytogenetic subgroup as those with high hyperdiploid (i.e., between 51 and 67 chromosomes), the COG defines this group as those with trisomy of chromosomes 4 and 10. Of note, subsequent UKALL analyses are likely to further refine their definition of this group as an indicator of good risk genetics [24]. Additionally, the use of hypodiploidy as an inclusion criterion for HR cytogenetic classification differs between the COG and UKALL groups. The COG considers all individuals with hypodiploidy (<43 chromosomes) as HR. UKALL considers two subsets of hypodiploidy as HR: “near haploidy” (<30 chromosomes) and “low hypodiploidy” (between 30 and 39 chromosomes). Thus, the difference reduces to the small subset of individuals between 40 and 42 chromosomes. TCF3-HLF positivity also contributes to UKALL’s HR definition. TCF3-HLF is indeed a very high-risk factor but is exceedingly rare and not routinely assessed in genetic testing algorithms.

We show that PI_COG_ can identify heterogeneity in outcome among the categorically defined RGs used in the current COG risk classification, suggesting that using continuous information may enhance traditional RG designation. This refinement of current RGs could offer further options for therapeutic interventions for certain subsets of patients. As outcomes continue to improve, the burden of treatment-related toxicity becomes an increasingly important consideration [25, 26]. For those with outstanding prognosis, a less intense chemotherapy regimen may help prevent the life-long complications of therapy, including cardiac disease, secondary cancers, decreased employment, and infertility [27]. For example, SR-Avg individuals who are PI_COG_ low risk could be considered for treatment de-intensification. In contrast, for those within the COG HR group with predicted outcome similar to the COG VHR group (e.g., PI_COG_ “high-risk”), innovative therapies could be considered to improve RFS.

Ideally, a fully independent external validation of PI_COG_ should be conducted with close attention to validation in minority demographic populations. Though Supplementary Fig. 8 shows good calibration and discrimination within each race/ethnicity subgroup and both sexes, a true external validation in minority populations is optimal for determining predictive performance. Prospective clinical trials could evaluate the PI_COG_’s efficacy as a clinical decision aid [28].

In addition to assessing the clinical performance of the PI_COG_, future research could assess further refinement with critical new prognostic factors. Modern clinical prediction models must be prepared to dynamically incorporate new discoveries and updated information [23]. For example, high-throughput sequencing (HTS) for MRD is more sensitive and easily standardized than standard flow cytometry and is a focus of current investigation in childhood ALL [29]. Updated models of ALL will also need to adapt to the growing importance of new genetic markers [30], or to improve the use of information from traditional ones [24]. A model-derived risk score, such as the PI_COG_, more readily allows the timely incorporation of such new information (such as HTS MRD and novel genetic subtypes) than do traditional risk stratification algorithms. Traditional risk stratification algorithms combining specific categories of many variables to construct RGs require extensive clinical knowledge regarding relationships between a new marker and other risk stratification variables to determine the appropriate algorithmic use for the new marker. Often, when a new prognostic marker is introduced, the first studies show only an association with outcome, with additional clinical knowledge following over the course of time. In contrast, when data becomes available on the new marker, established statistical methods parallel to those described in this paper can be applied to incorporate the new information into the model. Although model updating is nontrivial, the technology is available and could further strengthen the ability of the PI_COG_ to discriminate outcomes in groups of patients previously categorized together, presenting additional future opportunities to ask targeted questions.

This study has several strengths in addition to the size and data consistency of the study cohorts. The availability of D8 MRD, not routinely assessed by other groups, allowed incorporation of early disease response. Extensive prior studies of clinical and genomic variables as outcome predictors enabled this study to have predictor pre-specification instead of model-based selection, enhancing the applicability in external populations. Data-driven selection of PI_COG_ cutpoints to define RGs objectively optimizes outcome-based RG assignment. Several limitations also merit note. Certain patient subgroups (T-ALL, Down syndrome, Ph+) were not used to derive PI_COG_. The performance of PI_COG_ (or any PI) is unclear in small patient groups with limited data (e.g., Non-Hispanic/Other race or Ph-like). Future studies should assess calibration in such patient subgroups. Finally, because PI_COG_ relies on D29 MRD, only available at end-induction, it cannot be used to modify the first weeks of induction therapy.

In conclusion, contemporary ALL therapy relies on risk stratification but does not use all relevant rich and readily available data. The PI_COG_ showed a wide range of relapse risk within currently used RGs and thus may be useful as a clinical decision aid for future trials. Analogous efforts may have significant clinical value in other cancers.

### Supplementary information

Online Only Supplementary Material

## Data Availability

Children’s Oncology Group Data Sharing Statement: The Children’s Oncology Group Data Sharing policy describes the release and use of COG individual subject data for use in research projects in accordance with National Clinical Trials Network (NCTN) Program and NCI Community Oncology Research Program (NCORP) Guidelines. Only data expressly released from the oversight of the relevant COG Data and Safety Monitoring Committee (DSMC) are available to be shared. Data sharing will ordinarily be considered only after the primary study manuscript is accepted for publication. For phase 3 studies, individual-level de-identified datasets that would be sufficient to reproduce results provided in a publication containing the primary study analysis can be requested from the NCTN/NCORP Data Archive at https://nctn-data-archive.nci.nih.gov/. Data are available to researchers who wish to analyze the data in secondary studies to enhance the public health benefit of the original work and agree to the terms and conditions of use. For non-phase 3 studies, data are available following the primary publication. An individual-level de-identified dataset containing the variables analyzed in the primary results paper can be expected to be available upon request. Requests for access to COG protocol research data should be sent to: datarequest@childrensoncologygroup.org. Data are available to researchers whose proposed analysis is found by COG to be feasible and of scientific merit and who agree to the terms and conditions of use. For all requests, no other study documents, including the protocol, will be made available and no end date exists for requests. In addition to above, release of data collected in a clinical trial conducted under a binding collaborative agreement between COG or the NCI Cancer Therapy Evaluation Program (CTEP) and a pharmaceutical/biotechnology company must comply with the data sharing terms of the binding collaborative/contractual agreement and must receive the proper approvals.

## References

[1] Pui CH, Yang JJ, Hunger SP, Pieters R, Schrappe M, Biondi A (2015). Childhood acute lymphoblastic leukemia: progress through collaboration. J Clin Oncol.

[2] Hunger SP, Mullighan CG (2015). Acute lymphoblastic leukemia in children. N. Engl J Med.

[3] Salzer WL, Burke MJ, Devidas M, Chen S, Gore L, Larsen EC (2018). Toxicity associated with intensive postinduction therapy incorporating clofarabine in the very high-risk stratum of patients with newly diagnosed high-risk B-lymphoblastic leukemia: A report from the Children’s Oncology Group study AALL1131. Cancer.

[4] O’Connor D, Enshaei A, Bartram J, Hancock J, Harrison CJ, Hough R (2018). Genotype-specific minimal residual disease interpretation improves stratification in pediatric acute lymphoblastic leukemia. J Clin Oncol.

[5] Enshaei A, O’Connor D, Bartram J, Hancock J, Harrison CJ, Hough R (2020). A validated novel continuous prognostic index to deliver stratified medicine in pediatric acute lymphoblastic leukemia. Blood.

[6] Borowitz MJ, Devidas M, Hunger SP, Bowman WP, Carroll AJ, Carroll WL (2008). Clinical significance of minimal residual disease in childhood acute lymphoblastic leukemia and its relationship to other prognostic factors: a Children’s Oncology Group study. Blood.

[7] Raetz EA, Teachey DT (2016). T-cell acute lymphoblastic leukemia. Hematol Am Soc Hematol Educ Program.

[8] Maloney KW, Devidas M, Wang C, Mattano LA, Friedmann AM, Buckley P (2020). Outcome in children with standard-risk b-cell acute lymphoblastic leukemia: Results of children’s oncology group trial aall0331. J Clin Oncol.

[9] Larsen EC, Devidas M, Chen S, Salzer WL, Raetz EA, Loh ML (2016). Dexamethasone and high-dose methotrexate improve outcome for children and young adults with high-risk B-acute lymphoblastic leukemia: a report from children’s oncology group study AALL0232. J Clin Oncol.

[10] Angiolillo AL, Schore RJ, Kairalla JA, Devidas M, Rabin KR, Zweidler-McKay P (2021). Excellent outcomes with reduced frequency of vincristine and dexamethasone pulses in standard-risk B-lymphoblastic leukemia: results from Children’s Oncology Group AALL0932. J Clin Oncol.

[11] Burke MJ, Salzer WL, Devidas M, Dai Y, Gore L, Hilden JM (2019). Replacing cyclophosphamide/cytarabine/ mercaptopurine with cyclophosphamide/ etoposide during consolidation/delayed intensification does not improve outcome for pediatric B-cell acute lymphoblastic leukemia: A report from the COG. Haematologica.

[12] Steyerberg EW. Clinical prediction models: a practical approach to development, validation, and updating. 2nd ed. Springer Nature Switzerland, Cham, 2019.

[13] Harrell FE. Regression modeling strategies: with applications to linear models, logistic and ordinal regression, and survival analysis. 2nd ed. Springer Nature Switzerland, Cham, 2015.

[14] Royston P, Altman DG. External validation of a Cox prognostic model: principles and methods. BMC Med Res Methodol. 2013;13. 10.1186/1471-2288-13-33. 10.1186/1471-2288-13-33 PMC3667097 23496923

[15] McLernon DJ, Giardiello D, Van Calster B, Wynants L, van Geloven N, van Smeden M (2023). Assessing performance and clinical usefulness in prediction models with survival outcomes: practical guidance for Cox proportional hazards models. Ann Intern Med.

[16] Harrell Jr FE. rms: Regression Modeling Strategies. R package version 6.3-0, <https://CRAN.R-project.org/package=rms> (2022).

[17] Ishwaran H, Kogalur UB, Blackstone EH, Lauer MS (2008). Random survival forests. Ann Appl Stat.

[18] Fouodo CJK, König IR, Weihs C, Ziegler A, Wright MN (2018). Support vector machines for survival analysis with R. R J.

[19] Buehlmann P, Hothorn T (2007). Boosting algorithms: regularization, prediction and model fitting (with discussion). Stat Sci.

[20] Barrio I, Rodríguez-Alvarez MX, Meira-Machado L, Esteban C, Arostegui I (2017). Comparison of two discrimination indexes in the categorisation of continuous predictors in time-to-event studies. Sort.

[21] R Core Team. R: A language and environment for statistical computing. R Foundation for Statistical Computing, Vienna, Austria. URL https://www.R-project.org/ (2022).

[22] Chen Y, Millar JA. Machine learning techniques in cancer prognostic modeling and performance assessment. In: Frontiers of biostatistical methods and applications in clinical oncology. Springer Nature Singapore, Singapore 189721, 2017; 179–230.

[23] Winick N, Devidas M, Chen S, Maloney K, Larsen E, Mattano L (2017). Impact of initial CSF findings on outcome among patients with national cancer institute standard- and high-risk B-cell acute lymphoblastic leukemia: a report from the Children’s Oncology Group. J Clin Oncol.

[24] Enshaei A, Vora A, Harrison CJ, Moppett J, Moorman AV (2021). Defining low-risk high hyperdiploidy in patients with paediatric acute lymphoblastic leukaemia: a retrospective analysis of data from the UKALL97/99 and UKALL2003 clinical trials. Lancet Haematol.

[25] Mody R, Li S, Dover DC, Sallan S, Leisenring W, Oeffinger KC (2008). Twenty-five-year follow-up among survivors of childhood acute lymphoblastic leukemia: a report from the Childhood Cancer Survivor Study. Blood.

[26] Mulrooney DA, Hyun G, Ness KK, Bhakta N, Pui CH, Ehrhardt MJ (2019). The changing burden of late health outcomes in adult survivors of childhood acute lymphoblastic leukemia: a report from the St. Jude Lifetime cohort study. Lancet Haematol.

[27] Dixon SB, Chen Y, Yasui Y, Pui CH, Hunger SP, Silverman LB (2020). Reduced morbidity and mortality in survivors of childhood acute lymphoblastic leukemia: a report from the childhood cancer survivor study. J Clin Oncol.

[28] Cowley LE, Farewell DM, Maguire S, Kemp AM (2019). Methodological standards for the development and evaluation of clinical prediction rules: a review of the literature. Diagn. Progn Res.

[29] Wood B, Wu D, Crossley B, Dai Y, Williamson D, Gawad C (2018). Measurable residual disease detection by high-throughput sequencing improves risk stratification for pediatric B-ALL. Blood.

[30] Brady SW, Roberts KG, Gu Z, Shi L, Pounds S, Pei D (2022). The genomic landscape of pediatric acute lymphoblastic leukemia. Nat Genet.

