# Supplementary material for: Enhanced Risk Stratification for Children and Young Adults with B-Cell Acute Lymphoblastic Leukemia: A Children’s Oncology Group Report
Source: Leukemia. 2024 Feb 15;38(4):720–8. doi: 10.1038/s41375-024-02166-1 (PMC10997503; doi:10.1038/s41375-024-02166-1)
Supplement: Supplementary file 1 — Online Only Supplementary Material [file 41375_2024_2166_MOESM1_ESM.pdf]

Online Only Supplement

Table of Contents

Supplemental Methods *3*

Genetic Risk Group Definitions 3

CNS Status Definition 3

Deviation from Current AALL1732 HR Risk Stratification 3

Ph-Like Definition 4

Supplemental Tables *6*

Supplemental Table 1 6

Supplemental Table 2 7

Supplemental Table 3 8

Supplemental Table 4 9

Supplemental Table 5 9

Supplemental Table 6 11

Supplemental Table 7 11

Supplemental Table 8 12

Supplemental Table 9 12

Supplemental Table 10 13

Supplemental Table 11 13

Supplemental Table 12 14

Supplemental Table 13 14

Supplemental Table 14 15

Supplemental Table 15 16

Supplemental Table 16 16

Supplemental Table 17 17

Supplemental Table 18 17

Supplemental Figures *18*

Supplemental Figure 1 18

Supplemental Figure 2 19

Supplemental Figure 3 20

Supplemental Figure 4 21

Supplemental Figure 5 .... **22**
Supplemental Figure 6 .... **23**
Supplemental Figure 7 .... **24**
Supplemental Figure 8 .... **25**
References ***26***

Supplemental Methods

Genetic Risk Group Definitions

Unfavorable Risk Genetics (URG), Favorable Risk Genetics (FRG), and Intermediate Risk Genetics (IRG) were defined as follows:

- URG: Patients with hypodiploid ALL, *KMT2A* rearrangements, and/or intrachromosomal amplification of chromosome 21 (iAMP21) were classified as URG regardless of good risk (GR) cytogenetic factors (*ETV6::RUNX1* fusions and double trisomies of chromosome 4, 10 (DT)).
- FRG: Patients with *ETV6::RUNX1* fusions and/or DT with no high risk (HR) cytogenetic factors (hypodiploid ALL, *KMT2A* rearrangements, iAMP21) were classified as FRG.
- IRG: Those who were not classified as URG or FRG (confirmed no hypodiploid ALL, *KMT2A* rearrangements, iAMP21, *ETV6::RUNX1* fusions, or DT) were classified as IRG.
- Indeterminate: Certain patients could not be classified as URG, FRG, or IRG due to combinations of missing cytogenetic factors. Patients who are missing evaluation for hypodiploid ALL and/or *KMT2A* rearrangements with no other confirmed HR cytogenetic factors could not be assumed to be URG, FRG, or IRG due to the missing HR cytogenetic information. Patients without hypodiploid ALL, *KMT2A* rearrangements, and iAMP21 (confirmed no HR cytogenetic factors) who have no confirmed GR cytogenetic factors and are missing evaluation for *ETV6::RUNX1* fusions and/or DT could not be assumed to be FRG or IRG due to the missing GR cytogenetic information.

CNS Status Definition

CNS 1: In cerebral spinal fluid (CSF), absence of blasts on cytospin preparation, regardless of the number of white blood cells (WBCs).

CNS 2: In CSF, presence < 5/μL WBCs and cytospin positive for blasts, or traumatic lumbar puncture, > 5/μL WBCs, cytospin positive for blasts, but negative by Steinherz/Bleyer algorithm (see below):

CNS 2a: < 10/μL Red Blood Cells (RBCs); < 5/μL WBCs and cytospin positive for blasts;
CNS 2b: ≥ 10/μL RBCs; < 5/μL WBCs and cytospin positive for blasts; and
CNS 2c: ≥ 10/μL RBCs; ≥ 5/μL WBCs and cytospin positive for blasts but negative by Steinherz/Bleyer algorithm (see below).

CNS3: In CSF, presence of ≥ 5/μL WBCs and cytospin positive for blasts and/or clinical signs of CNS leukemia:

CNS 3a: < 10/µL RBCs; ≥ 5/µL WBCs and cytospin positive for blasts;
CNS 3b: ≥ 10/µL RBCs, ≥ 5/µL WBCs and positive by Steinherz/Bleyer algorithm (see below);
CNS 3c: Clinical signs of CNS leukemia (such as facial nerve palsy, brain/eye involvement or hypothalamic syndrome).

<u>Method of evaluating initial traumatic lumbar punctures (Steinherz/Bleyer algorithm definition):</u>
If the patient has leukemic cells in the peripheral blood and the lumbar puncture is traumatic and contains ≥ 5 WBC/μL and blasts, the following Steinherz/Bleyer algorithm should be used to distinguish between CNS2 and CNS3 disease:

$$\frac{CSF\ WBC}{CSF\ RBC} > 2 \times \frac{Blood\ WBC}{Blood\ RBC}$$

A patient with CSF WBC ≥ 5/μL blasts, whose CSF WBC/RBC is 2X greater than the blood WBC/RBC ratio, has CNS disease at diagnosis.

Deviation from Current AALL1732 HR Risk Stratification

We took minor deviations in our COG retrospective risk classification of High Risk (HR) and Very High Risk (VHR) patients from AALL1732's current risk classification schema. On AALL1732, Philadelphia-Like (Ph-Like) NCI HR patients (CRLF2/JAK lesions, ABL class fusion, or another Ph-Like gene expression profile) were separated into their own risk group. We did not separate individuals into this risk stratification category as the

database-available Ph-like information is not uniformly collected. Additionally, in AALL1732, end-of-consolidation (EOC) MRD was used to define the VHR group. This was a primarily an inclusion criterion for another trial rather than a strong prognostic difference. Therefore, use of EOC MRD was removed for the retrospective risk classification.

Ph-Like Definition

Philadelphia Chromosome-Like (Ph-like) ALL: Ph-like positive patients were identified during induction by Low Density Array (LDA). Additional testing was used to identify those patients with ABL-class fusions as well as those with CRLF2/JAK pathway fusions. Further details as decribed.[1,2]

Prognostic Index Cutpoint Detection

Determination of cutpoints which define the "Low", "Standard", "Intermediate", and "High" risk groups of the PI_{COG} uses the cutpoint detection method for continuous variables proposed by Barrio et al.[3] The following is a summary of this proposed method.

Discriminative ability of a Cox model is quantified by the probability of concordance (C):

$$\mathrm{C} = \Pr(\tilde{T}_i > \tilde{T}_j | T_i > T_j)$$

where for subject i, $\tilde{T}_i$ is the model-predicted survival time and T_i is the observed survival time. There are two common ways to estimate C. For individual i, let t_i be the event time, c_i be the censoring time, $y_i = \min(t_i, c_i)$, $\hat{\eta}_i$ be the linear predictor from the estimated Cox model, and $\delta_i = I(t_i \leq c_i)$.

1. The C-Index

$$C - Index = \frac{\sum\sum_{i<j}\{I(y_i < y_j)I(\hat{\eta}_i < \hat{\eta}_j)\delta_i + I(y_j < y_i)I(\hat{\eta}_j < \hat{\eta}_i)\delta_j\}}{\sum\sum_{i<j}\{I(y_i < y_j)\delta_i + I(y_j < y_i)\delta_j\}}$$

2. The Concordance Probability Estimator (CPE)

$$\mathrm{CPE} = \frac{2}{N(N-1)}\sum\sum_{i<j}\left\{\frac{I(\hat{\eta}_i < \hat{\eta}_j)}{1+\exp(\hat{\eta}_i - \hat{\eta}_j)} + \frac{I(\hat{\eta}_j < \hat{\eta}_i)}{1+\exp(\hat{\eta}_i - \hat{\eta}_j)}\right\}$$

The C-Index is the most reported estimator in medical literature, and hence was the discrimination index reported in this paper. However, the C-Index is biased, and the CPE was originally proposed as an asymptotically unbiased alternative.

Let X be the continuous PI_{COG} which needs to be divided into four risk groups using cutpoints. We wish to categorize X in a way such that the resulting risk groups give us the best (maximum) discrimination as measured by the concordance probability C with respect to risk of relapse when considered as the single variable in a Cox proportional hazards model. In the context of our application, Barrio et al. give notation for this maximization problem as follows:

Given $k = 3$ cutpoints, we will categorize X into $k + 1 = 4$ intervals. Denote the categorized variable as X_{Cat_3} which takes values {0,1,2,3} corresponding to "Low", "Standard", "Intermediate", and "High" risk groups, respectively. Let $x = [x_1, x_2, x_3]^T$ be the vector of cutpoints that categorize X. The task is to find the cutpoint vector x such that the concordance probability of the following Cox model is maximized:

$$h(t|X_{Cat_3}) = h_0(t)\exp\left(\sum_{q=1}^{3}\beta_q I(X_{Cat_3} = q)\right)$$

where $I(X_{Cat_3} = q)$ is an indicator function taking on the value 1 when $X_{Cat_3} = q$ and 0 else. To compare the concordance probability of two Cox models using two different sets of cutpoints for X (say $h^*(t|X_{Cat_3})$ and

$h^{\dagger}\left(t \middle| X_{Cat_3}\right)$ corresponding to using two different vectors of cutpoints x^{*} and $x^{\dagger}$, respectively), Barrio et al. propose estimating C for each model using either the C-Index or the CPE. In this paper, we chose to use the asymptotically unbiased CPE. Then, whichever of CPE^{*} and $\text{CPE}^{\dagger}$ is larger indicates that the corresponding cutpoints generate the model with better discriminative ability.

An exhaustive search of all possible vectors of cutpoints $x = [x_1, x_2, x_3]^T$ is computationally prohibitive. Therefore, Barrio et al. suggest using an algorithmic search and provide two different commonly used algorithm options: the *AddFor* algorithm and the *Genetic* algorithm. We do not go into details of these search algorithms here but direct the interested reader to Barrio et al.[3] We chose to use the *Genetic* algorithm due to good performance in the simulation study presented by the authors. Therefore, the vector of cutpoints x with the maximum CPE as identified by the *Genetic* algorithm are the cutpoints that we use to define the risk groups for the PI_{COG}.

The steps above are implemented in R Statistical Software via the *CatPredi* package by Barrio et al.[4]

Supplemental Tables

Supplemental Table 1. Candidate covariates for the new PI_{COG} model.

Variable Considered	Degrees of Freedom needed (if covariate in model)	Include?	Notes
Main Terms			
Original Protocol	3	X	Minimal differences in effect of treatment
NCI Risk	1	X	Included by using WBC and Age
Sex	1	X	Not a known prognostic factor for primary ALL outcomes
Race	6	X	Poor proxy measurement, data quality concerns
Ethnicity	2	X	Poor proxy measurement, data quality concerns
BMI	1	X	Concerns for accuracy of BMI and consistency of weight/height collection
WBC	1	✓	
Age	1	✓	
CNS Status	2	✓	
Testicular Leukemia	2	X	Very small subset of patients (n=57) with well-defined treatment approaches
Cyto-GR	1		
ETV6/RUNX1		✓	
DT		✓	
Cyto-HR	1		
KMT2A		✓	
Hypodiploid		✓	
i*AMP*21		✓	
Ph-Like	1	X	Sparse (77.9% of patients not tested)
CRLF2	1	X	Sparse (77.2% of patients not tested)
D8 MRD	3	✓	
D29 MRD	3	✓	
Interaction Terms			
D29 MRD:Cyto-GR	1	X	Contribution of interactions to the full multivariable model were jointly tested using a 13 degree of freedom likelihood ratio test (p=0.06)
D29 MRD:Cyto-HR	1	X	
Age:Cyto-GR	3	X	
Age:Cyto-HR	3	X	
Age:WBC	3	X	
WBC:Cyto-GR	1	X	
WBC:Cyto-HR	1	X	

Supplemental Table 2. UK Prognostic Index (PI) external validation steps (adapted from Royston and Altman, 2013).[5]

Step	Action	Information required	Sufficient Information to conduct step?
(1) Regression of outcome on PI in external validation data	Univariable cox regression of RFS on UK PI calculated for COG external validation data to obtain the overall calibration slope (the regression coefficient associated with this model), formal hypothesis test of the null hypothesis that the overall calibration slope is equal to one (ideal) obtained by fitting the univariable model with the UK PI as an offset	Published set of regression coefficients	Yes
(2) Check model fit in external validation data	Refit the UK PI model in the COG external validation data and include the published UK PI as an offset to obtain formal test of the null hypothesis that the difference between derived and published coefficients is equal to zero (ideal)	Published set of regression coefficients	Yes
(3) Report PI discrimination in external validation data metrics	Calculate the concordance index associated with the published UK PI calculated for the COG external validation data	Published set of regression coefficients	Yes
(4) Visualize Kaplan-Meier curves within PI-defined risk groups	Calculate Kaplan-Meier curves for COG external validation data stratified by published UK PI-defined risk groups	Published set of regression coefficients and Kaplan-Meier curves in original development data for comparison	Yes*
(5) Report hazard ratios associated with each published risk group	Univariable cox regression of RFS on UK PI risk groups calculated for COG external validation data to obtain a hazard ratio for each risk group, compare to the same hazard ratios in the UK PI development data	Published set of regression coefficients and hazard ratio associated with each risk group in original development data for comparison	No - hazard ratios across risk groups in development data unavailable
(6) Assess calibration in the external validation data	Compared UK PI model-predicted mean survival curves for the COG external validation data to observed Kaplan-Meier curves in the external validation data	Published set of regression coefficients, Kaplan-Meier curves in original development data, and an estimate of the baseline survival function in the original development data	No – estimate of baseline survival function from development data unavailable

*Kaplan-Meier summaries needed to reconstruct survival curves within risk groups for UK PI development data were unavailable. Therefore, Kaplan-Meier curves for the UK PI-defined risk groups in the COG external validation data are reported here and can be compared to the Kaplan-Meier curves in Enshaei et al., 2020 as described in Royston and Altman, 2013.[5]

Supplemental Table 3. Summary and results of machine learning benchmark study.

Model	Cox Proportional Hazards Model	Random Forest Model	Survival Nonlinear Support Vector Machine (SVM)*	Gradient Boosted Cox Model
High Interpretability?	x			
Flexible Functional Relationship?		x	x	
Flexible Interactions?		x	x	
Ensemble?		x		x
Preserves Continuity?	x		x	x
C-Index	0.752	0.749	0.737	0.751
R Package	survival	randomForestSRC[6]	Survivalsvm[7]	Mboost[8]

Note: Benchmarking study implemented using the *mlr* framework in R.[9]
*Computation (time and memory) of survival SVM is currently excessive as sample size increases.[10] Frequently, heavy algorithms are trained on a representative subsample of the data to aid computation. This survival SVM was fit on a random sub-sample of n=1,000.

Supplemental Table 4. Retrospective risk stratification algorithm according to the current generation of standard risk COG clinical trials. AALL1731: NCI Non-DS SR B-ALL Patients (Excluding Patients with Steroid Pretreatment1, CNS3, or Testicular Leukemia)

Prognostic Factor	SR-Favorable		SR-Average			SR-High	
CNS	1/2	1/2	1/2	1	2	1/2	1/2
Cytogenetics	Fav	Fav	DT	Neut	Neut	Unfav	Any
Day 8 PB MRD	<1	≥1	Any	Any	Any	Any	Any
EOI MRD (%)	<0.01	<0.01	0.01 to <0.1	<0.01	<0.01	<0.01	≥0.01
EOC MRD (%)	n/a	n/a	n/a	n/a	n/a	n/a	<1%

Supplemental Table 5. Retrospective risk stratification algorithm according to the current generation of high risk COG clinical trials. Retrospective Classification According to AALL1732: NCI HR B-ALL Patients and NCI SR B-ALL Patients with CNS3, Testicular Leukemia, or Steroid Pretreatment

Prognostic Variable	HR-Favorable		High Risk		Very High Risk
NCI Risk Group	HR < 10 yr	SR	HR (except HR-Fav)	HR	HR
CNS/Testicular Leukemia	CNS1, no testicular leukemia	CNS3, testicular leukemia, or steroid pretreatment	Any	Any	Any
Cytogenetics	Fav	Any	Any	Fav/Neut	Unfav
EOI MRD (%)	<0.01	Any	<0.01	≥0.01	0.01

Supplemental Table 6. Patient Characteristics of the Post-Induction Relapse-Free Survival Cohort (Figure 1, n=15202)*

	Testing (n=4100)	Training (n=11102)	Total (n=15202)
Age in years, median (range)	4.83 (1.0, 30.8)	4.58 (1.0, 30.8)	4.58 (1.0, 30.8)
Sex (%)			
Female	1929 (47.1)	5064 (45.6)	6993 (46.0)
Male	2171 (53.0)	6038 (54.4)	8209 (54.0)
NCI Risk (%)			
SR	2748 (67.0)	8464 (76.2)	11212 (73.8)
HR	1352 (33.0)	2628 (23.8)	3990 (26.3)
WBC x 1000/µl, median (range)	8.60 (0.30, 1148.5)	8.00 (0.1, 5800.0)	8.20 (0.1, 5800.0)
CNS (%)			
CNS1	3646 (88.9)	9983 (89.9)	13629 (89.7)
CNS2	403 (9.8)	1029 (9.3)	1432 (9.4)
CNS3	51 (1.2)	90 (0.8)	141 (0.9)
Race (self-declared) (%)			
Asian	177 (4.3)	515 (4.6)	692 (4.6)
Black	276 (6.7)	633 (5.7)	909 (6.0)
White	3119 (76.1)	8145 (73.4)	11264 (74.1)
Other	42 (1.0)	243 (2.2)	285 (1.9)
Ethnicity (self-declared) (%)			
Hispanic	999 (24.4)	2699 (24.3)	3698 (24.3)
Non-Hispanic	2935 (71.6)	7910 (71.3)	10845 (71.3)
Unknown	166 (4.1)	493 (4.4)	525 (3.5)
Cytogenetics (%)			
ETV6::RUNX1	1050 (25.6)	3070 (27.7)	4120 (27.1)
Double Trisomy	980 (23.9)	2753 (24.8)	3733 (24.6)
iAMP21	97 (2.4)	258 (2.3)	355 (2.3)
Hypodiploidy	66 (1.6)	146 (1.3)	212 (1.4)
Ph-like†	116	341	457
KMT2Ar	77 (1.9)	149 (1.3)	226 (1.5)
PB MRD Day 8 (%)			
< 0.01%	785 (19.2)	2587 (23.3)	3372 (22.2)
0.01-<0.1%	1041 (25.4)	3011 (27.1)	4052 (26.7)
0.1 to < 1.0%	1252 (30.5)	3227 (29.1)	4479 (29.5)
>/= 1.0%	1022 (24.9)	2277 (20.5)	3299 (21.7)
BM MRD Day 29 (%)			
< 0.01%	3178 (77.5)	8926 (80.4)	12104 (79.6)
0.01-<0.1%	459 (11.2)	1149 (10.4)	1608 (10.6)
0.1 to < 1.0%	334 (8.2)	739 (6.7)	1073 (7.1)
>/= 1.0%	129 (3.2)	288 (2.6)	417 (2.7)
Event type (%)			
None	3513 (85.7)	10040 (90.4)	13553 (89.2)
Relapse	482 (11.8)	863 (7.8)	1345 (8.9)
Remission Death	68 (1.7)	151 (1.4)	219 (1.4)
Second Malignant Neoplasm	37 (0.9)	48 (0.4)	85 (0.6)

*See CONSORT diagram in Figure 1 for determination of the post-induction relapse-free survival cohort used in model development and numeric validation. Abbreviations: MRD, minimal residual disease; Race "Other" includes: Native Hawaiian/other Pacific Islander, American Indian or Alaska Native, and Multiple Races

†Ph-Like testing was not conducted uniformly on all patients, therefore percentages are omitted as they may not indicate a representative proportion

Supplemental Table 7. Added predictive value of transformed D8 MRD to the PI_{UKALL} (C=0.736).

Variable	Coefficient	HR (95% CI)	P-value
τ(D29 MRD)	-0.123	0.88 (0.87-0.90)	<0.001
τ(D8 MRD)	-0.040	0.96 (0.94-0.98)	<0.001
FRG	-0.877	0.42 (0.37-0.47)	<0.001
URG	0.755	2.13 (1.84-2.46)	<0.001
WBC_{log}	0.154	1.17 (1.12-1.21)	<0.001

Supplemental Table 8. Internal validation summaries for the Cox-Proportional hazards model from which the PI_{COG} was derived (B=1,000 bootstrap resamples).

Measurement	Original Index	Training	Test	Optimism	Corrected Index	Percent Optimism	B
C-Index	0.7551	0.7559	0.7542	0.0017	0.7534	0.23	1000
Dxy	0.5101	0.5118	0.5083	0.0035	0.5066	0.68	1000
R2	0.0968	0.0978	0.0960	0.0018	0.0951	1.86	1000
Slope	1.0000	1.0000	0.9908	0.0092	0.9908	0.92	1000
D	0.0490	0.0495	0.0486	0.0010	0.0480	2.04	1000
U	-0.0001	-0.0001	0.0001	-0.0002	0.0001	200	1000
Q	0.0491	0.0497	0.0485	0.0011	0.0480	2.24	1000
g	0.9103	0.9154	0.9062	0.0092	0.9011	1.01	1000

Note: Measurement: a summary statistic measuring the performance of the model (D, U, Q, and g included to allow comparison to possible future models using more precise log-likelihood based statistics); Original Index: the summary statistic in the original model fit; Training: the average summary statistic in training resamples; Testing: the average summary statistic in testing resamples; Optimism: Training-Testing; Corrected Index: Original Index - Optimism; Percent Optimism: percent change between Original Index and Corrected Index; B: Number of successful bootstrap repetitions.[11]

C-Index: Concordance Index; Dxy: Somer's Rank Correlation; R2: Nagelkerke R^2; Slope: Overall calibration slope; D: Discrimination Index; U: Unreliability Index; Q: Logarithmic accuracy score; g: g-Index on scale of linear predictor.[12]

Supplemental Table 9. COG retrospective risk classification results for full analysis population (%).

Original Study	SR Favorable (5 Yr. RFS=96.69%)	SR Fav/Avg* (96.08%)	SR Average (93.31%)	SR High (82.70%)	HR Favorable (96.33%)	HR (81.80%)	VHR (53.62%)	NA (88.69%)	Total
AALL0232	6 (0.12)	5 (0.44)	6 (0.14)	4 (0.15)	257 (40.79)	2427 (38.62)	101 (42.62)	94 (12.75)	2900
AALL0331	1197 (23.09)	1089 (96.46)	1470 (33.78)	962 (36.37)	0 (0.00)	114 (1.81)	0 (0.00)	267 (36.23)	5099
AALL0932	3981 (76.78)	27 (2.39)	2862 (65.76)	1672 (63.21)	0 (0.00)	38 (0.60)	0 (0.00)	196 (26.59)	8776
AALL1131	1 (0.02)	8 (0.71)	14 (0.32)	7 (0.26)	373 (59.21)	3705 (58.96)	136 (57.38)	180 (24.42)	4424
Total	5185 (24.46)	1129 (5.33)	4352 (20.53)	2645 (12.48)	630 (2.97)	6284 (29.64)	237 (1.12)	737 (3.48)	21199

*SR Favorable/Average (SR Fav/Avg) are individuals who were either SR Favorable or SR Average by other factors, but were missing D8 MRD to distinguish and were as such kept track of in an internal group.

Supplemental Table 10. 5-year Disease-Free survival (DFS) estimation for subgroups by COG retrospective and COG Prognostic Index risk classifications in the combined training/testing data.

	COG PI Classification			
**COG Risk Classification**	**Low**	**Standard**	**Intermediate**	**High**
SR Fav	0.966 (0.003)	0.927 (0.015)	--	--
SR Avg	0.959 (0.008)	0.934 (0.005)	0.899 (0.019)	--
SR High	0.924 (0.052)	0.894 (0.013)	0.826 (0.013)	0.721 (0.022)
HR Fav	0.977 (0.010)	0.964 (0.014)	--	--
HR	0.955 (0.014)	0.901 (0.010)	0.841 (0.010)	0.652 (0.015)
VHR	--	--	--	0.534 (0.044)

Note:
Empty cells indicate insufficient sample size for reliable estimation (< 25 patients).
Patients in SR-Fav/Avg (Supplemental Table 9) are missing MRD8, as such they are not represented in this table.

Supplemental Table 11. 5-year Overall Survival (OS) estimation for subgroups by COG retrospective and COG Prognostic Index risk classifications in the combined training/testing data.

	COG PI Classification			
**COG Risk Classification**	**Low**	**Standard**	**Intermediate**	**High**
SR Fav	0.991 (0.001)	0.993 (0.005)	--	--
SR Avg	0.991 (0.004)	0.978 (0.003)	0.959 (0.012)	--
SR High	0.950 (0.049)	0.964 (0.008)	0.932 (0.008)	0.872 (0.017)
HR Fav	0.991 (0.006)	0.994 (0.006)	--	--
HR	0.983 (0.008)	0.958 (0.007)	0.908 (0.008)	0.803 (0.012)
VHR	--	--	--	0.656 (0.042)

Note:
Empty cells indicate insufficient sample size for reliable estimation (< 25 patients).
Patients in SR-Fav/Avg (Supplemental Table 9) are missing MRD8, as such they are not represented in this table.

Supplemental Table 12. Sample sizes (%) for subgroups by COG risk and COG Prognostic Index classification in the training data.

	COG PI Classification				
**COG Risk Classification**	**Low (5 Yr. RFS=96.99%)**	**Standard (93.07%)**	**Intermediate (85.82%)**	**High (66.91%)**	**Total**
SR Fav	3691 (94.52%)	214 (5.48%)	0 (0.00%)	0 (0.00%)	3905
SR Avg	477 (16.90%)	2176 (77.11%)	169 (5.99%)	0 (0.00%)	2822
SR High	20 (1.25%)	498 (31.24%)	759 (47.62%)	317 (19.89%)	1594
HR Fav	160 (60.84%)	103 (39.16%)	0 (0.00%)	0 (0.00%)	263
HR	183 (7.51%)	658 (27.01%)	847 (34.77%)	748 (30.71%)	2436
VHR	0 (0.00%)	0 (0.00%)	0 (0.00%)	81 (100.00%)	81
Total	4531	3649	1775	1146	11101

Supplemental Table 13. 5-year Relapse-Free Survival (RFS) estimation for subgroups by COG retrospective and COG Prognostic Index risk classifications in the training data.

	COG PI Classification			
**COG Risk Classification**	**Low**	**Standard**	**Intermediate**	**High**
SR Fav	0.972 (0.003)	0.914 (0.020)	--	--
SR Avg	0.962 (0.009)	0.943 (0.005)	0.894 (0.025)	--
SR High	--	0.906 (0.014)	0.852 (0.014)	0.736 (0.027)
HR Fav	0.975 (0.013)	0.959 (0.020)	--	--
HR	0.952 (0.017)	0.910 (0.012)	0.855 (0.012)	0.649 (0.018)
VHR	--	--	--	0.597 (0.056)

Note:
Empty cells indicate insufficient sample size for reliable estimation (< 25 patients).
Patients in SR-Fav/Avg (Supplemental Table 9) are missing MRD8, as such they are not represented in this table.

Supplemental Table 14. 5-year Disease-Free survival (DFS) estimation for subgroups by COG retrospective and COG Prognostic Index risk classifications in the training data.

	COG PI Classification			
**COG Risk Classification**	**Low**	**Standard**	**Intermediate**	**High**
SR Fav	0.969 (0.003)	0.914 (0.020)	--	--
SR Avg	0.962 (0.009)	0.940 (0.005)	0.886 (0.026)	--
SR High	--	0.902 (0.014)	0.849 (0.014)	0.725 (0.027)
HR Fav	0.968 (0.014)	0.959 (0.020)	--	--
HR	0.952 (0.017)	0.907 (0.012)	0.844 (0.013)	0.639 (0.018)
VHR	--	--	--	0.597 (0.056)

Note:
Empty cells indicate insufficient sample size for reliable estimation (< 25 patients).
Patients in SR-Fav/Avg (Supplemental Table 9) are missing MRD8, as such they are not represented in this table.

Supplemental Table 15. 5-year Overall Survival (OS) estimation for subgroups by COG retrospective and COG Prognostic Index risk classifications in the training data.

	COG PI Classification			
**COG Risk Classification**	**Low**	**Standard**	**Intermediate**	**High**
SR Fav	0.991 (0.002)	0.993 (0.007)	--	--
SR Avg	0.988 (0.005)	0.980 (0.003)	0.958 (0.016)	--
SR High	--	0.964 (0.009)	0.944 (0.009)	0.874 (0.020)
HR Fav	0.987 (0.009)	1.000 (0.000)*	--	--
HR	0.983 (0.009)	0.954 (0.008)	0.903 (0.010)	0.795 (0.015)
VHR	--	--	--	0.710 (0.053)

Note:
Empty cells indicate insufficient sample size for reliable estimation (< 25 patients).
Patients in SR-Fav/Avg (Supplemental Table 9) are missing MRD8, as such they are not represented in this table.
*No events fall in this subgroup, hence Greenwood's formula for the standard error of the Kaplan-Meier estimator evaluates to zero.

Supplemental Table 16. Sample sizes (%) for subgroups by COG risk and COG Prognostic Index classification in the testing data.

COG Risk Classification	COG PI Classification<br>Low<br>(5 Yr. RFS=96.32%)	Standard<br>(91.25%)	Intermediate<br>(82.96%)	High<br>(66.93%)	Total
SR Fav	1055 (90.02%)	117 (9.98%)	0 (0.00%)	0 (0.00%)	1172
SR Avg	155 (16.23%)	687 (71.94%)	113 (11.83%)	0 (0.00%)	955
SR High	8 (1.48%)	171 (31.61%)	230 (42.51%)	132 (24.40%)	541
HR Fav	59 (44.70%)	73 (55.30%)	0 (0.00%)	0 (0.00%)	132
HR	60 (4.85%)	332 (26.86%)	440 (35.60%)	404 (32.69%)	1236
VHR	0 (0.00%)	0 (0.00%)	0 (0.00%)	64 (100.00%)	64
Total	1337	1380	783	600	4100

Supplemental Table 17. 5-year Relapse-Free Survival (RFS) estimation for subgroups by COG retrospective and COG Prognostic Index risk classifications in the testing data.

COG Risk Classification	COG PI Classification<br>Low	Standard	Intermediate	High
SR Fav	0.963 (0.006)	0.953 (0.021)	--	--
SR Avg	0.957 (0.017)	0.919 (0.011)	0.916 (0.027)	--
SR High	--	0.880 (0.025)	0.765 (0.029)	0.721 (0.040)
HR Fav	1.000 (0.000)*	0.971 (0.020)	--	--
HR	0.963 (0.026)	0.889 (0.018)	0.841 (0.018)	0.683 (0.024)
VHR	--	--	--	0.446 (0.068)

Note:
Empty cells indicate insufficient sample size for reliable estimation (< 25 patients).
Patients in SR-Fav/Avg (Supplemental Table 9) are missing MRD8, as such they are not represented in this table.
*No events fall in this subgroup, hence Greenwood's formula for the standard error of the Kaplan-Meier estimator evaluates to zero.

Supplemental Table 18. 5-year Disease-Free survival (DFS) estimation for subgroups by COG retrospective and COG Prognostic Index risk classifications in the testing data.

	COG PI Classification			
**COG Risk Classification**	**Low**	**Standard**	**Intermediate**	**High**
SR Fav	0.958 (0.006)	0.953 (0.021)	--	--
SR Avg	0.951 (0.018)	0.918 (0.011)	0.916 (0.027)	--
SR High	--	0.875 (0.026)	0.754 (0.029)	0.708 (0.040)
HR Fav	1.000 (0.000)*	0.971 (0.020)	--	--
HR	0.963 (0.026)	0.889 (0.018)	0.834 (0.018)	0.676 (0.024)
VHR	--	--	--	0.446 (0.068)

Note:
Empty cells indicate insufficient sample size for reliable estimation (< 25 patients).
Patients in SR-Fav/Avg (Supplemental Table 9) are missing MRD8, as such they are not represented in this table.
*No events fall in this subgroup, hence Greenwood's formula for the standard error of the Kaplan-Meier estimator evaluates to zero.

Supplemental Table 19. 5-year Overall Survival (OS) estimation for subgroups by COG retrospective and COG Prognostic Index risk classifications in the testing data.

	COG PI Classification			
**COG Risk Classification**	**Low**	**Standard**	**Intermediate**	**High**
SR Fav	0.991 (0.003)	0.991 (0.009)	--	--
SR Avg	1.000 (0.000)*	0.971 (0.007)	0.963 (0.018)	--
SR High	--	0.964 (0.014)	0.895 (0.021)	0.862 (0.030)
HR Fav	1.000 (0.000)*	0.986 (0.014)	--	--
HR	0.982 (0.018)	0.964 (0.011)	0.916 (0.014)	0.817 (0.020)
VHR	--	--	--	0.581 (0.067)

Note:
Empty cells indicate insufficient sample size for reliable estimation (< 25 patients).
Patients in SR-Fav/Avg (Supplemental Table 9) are missing MRD8, as such they are not represented in this table.
*No events fall in this subgroup, hence Greenwood's formula for the standard error of the Kaplan-Meier estimator evaluates to zero.

Supplemental Figures

Supplemental Figure 1. Kaplan-Meier curves for relapse-free survival probability within each PI_{UKALL}-defined risk group for the combined RFS cohorts and corresponding risk table.

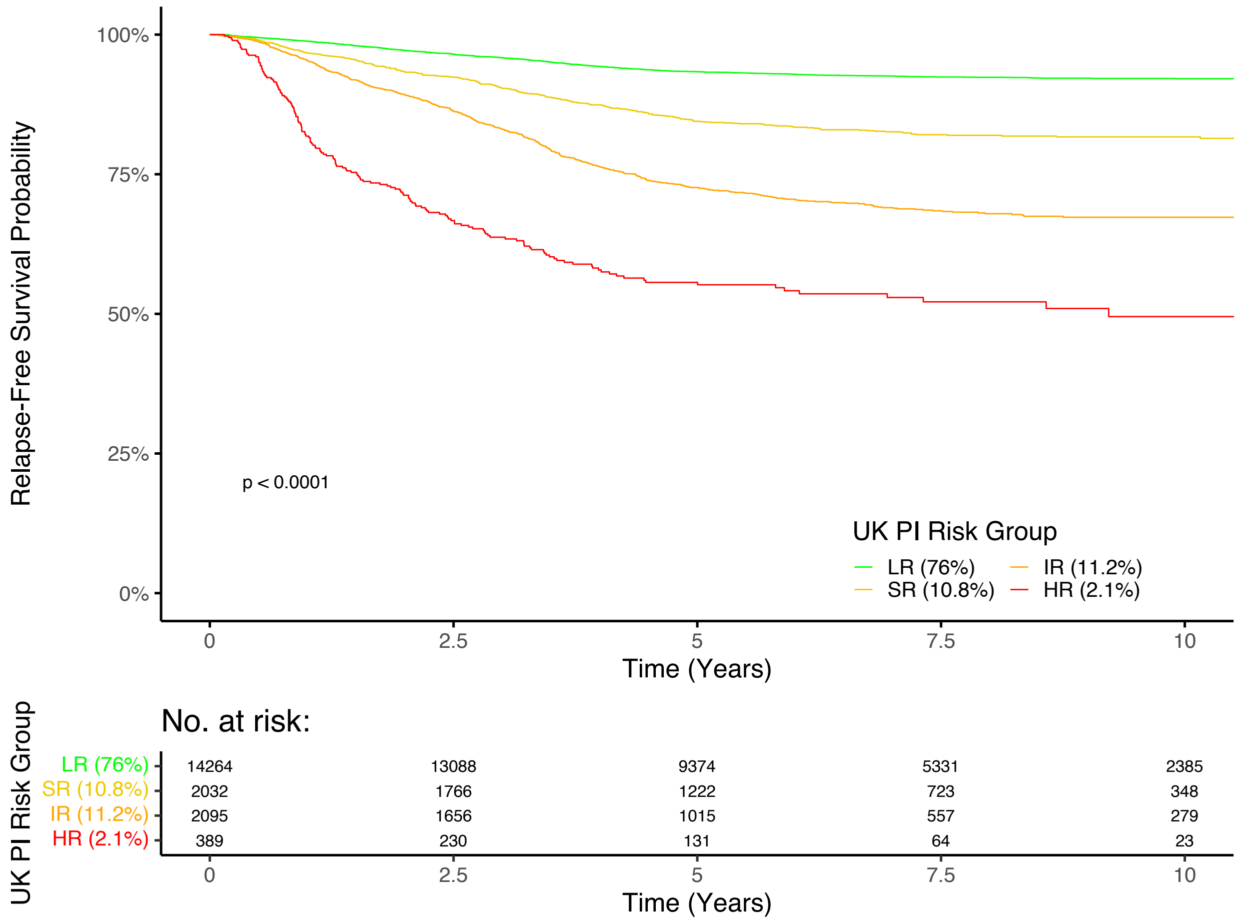

Supplemental Figure 2. Density plots of the distributions of the PI_{UKALL} and the PI_{COG} stratified by NCI risk group for the full analysis population. A) PI_{UKALL} distribution stratified by NCI standard risk (SR) and high risk (HR) shown in blue. B) PI_{COG} distribution stratified by NCI SR and HR shown in yellow.

A)

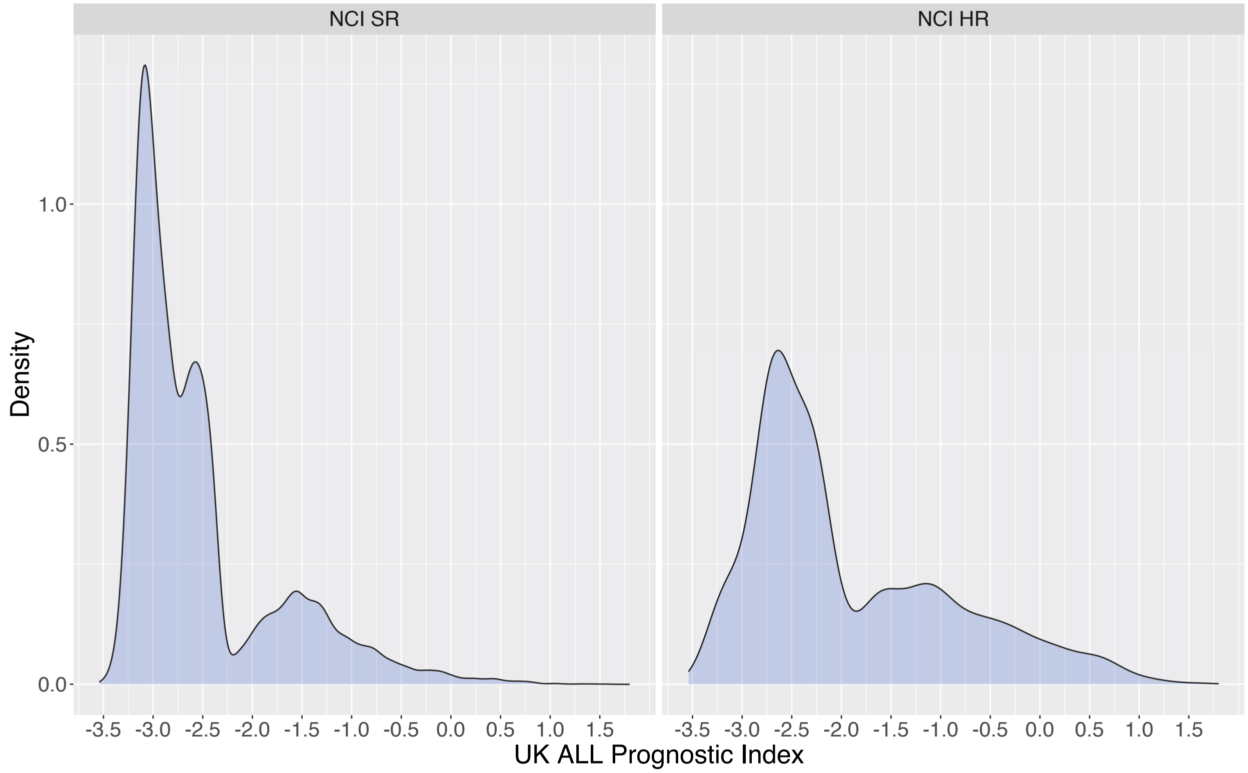

B)

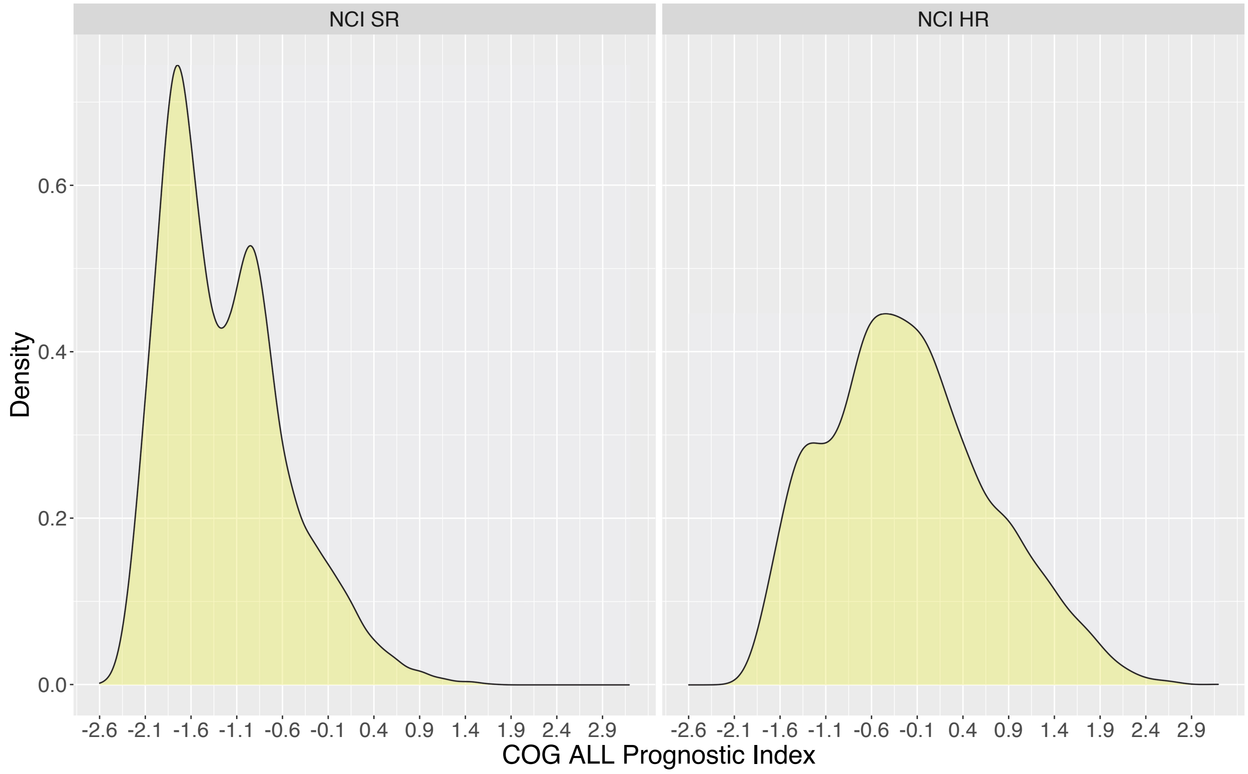

Supplemental Figure 3. Diagnostic plots used to check the assumptions of the Cox model developed on training data to derive the PI_{COG}. A) Plots of the scaled Schoenfeld residuals over time assess the proportional hazards (PH) assumption. Loess curves are shown in blue with 95% confidence bands. B) Delta-Beta (dfbeta) residuals visualize patients with high influence on coefficient estimation. Red horizontal dashed line indicates a residual of zero.

A)

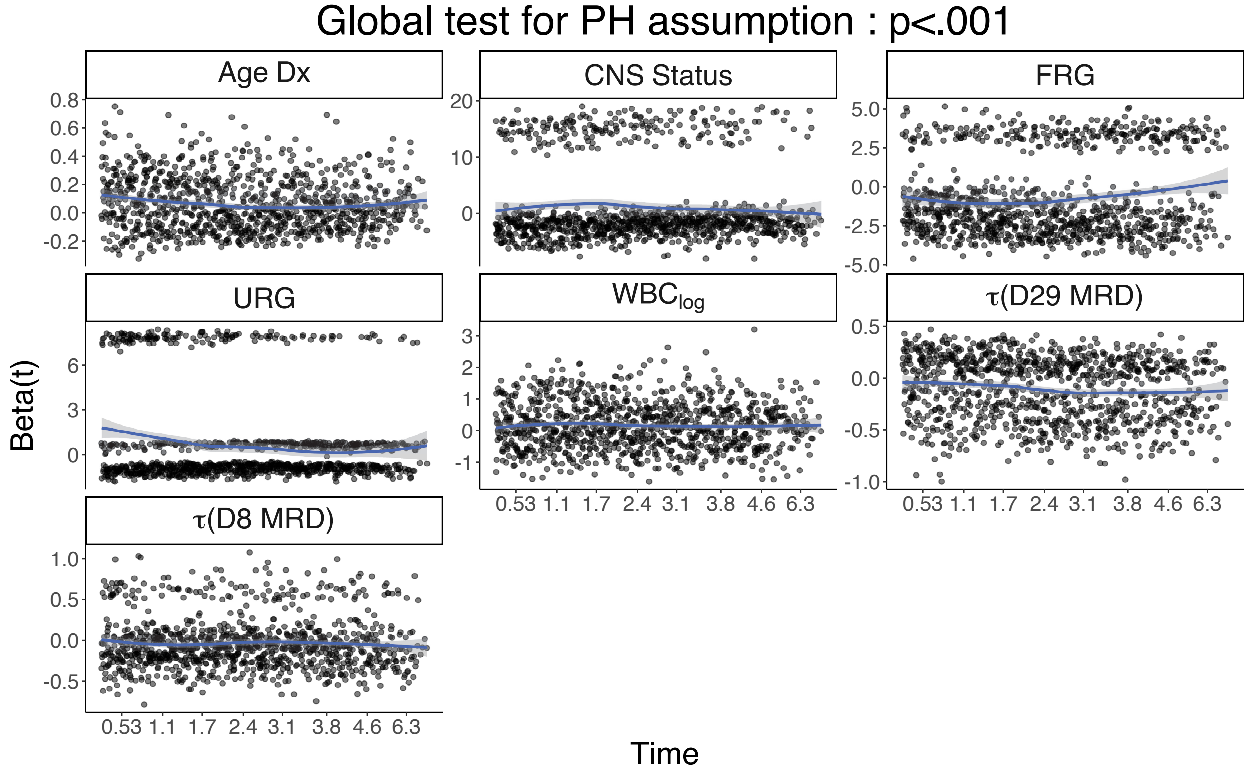

B)

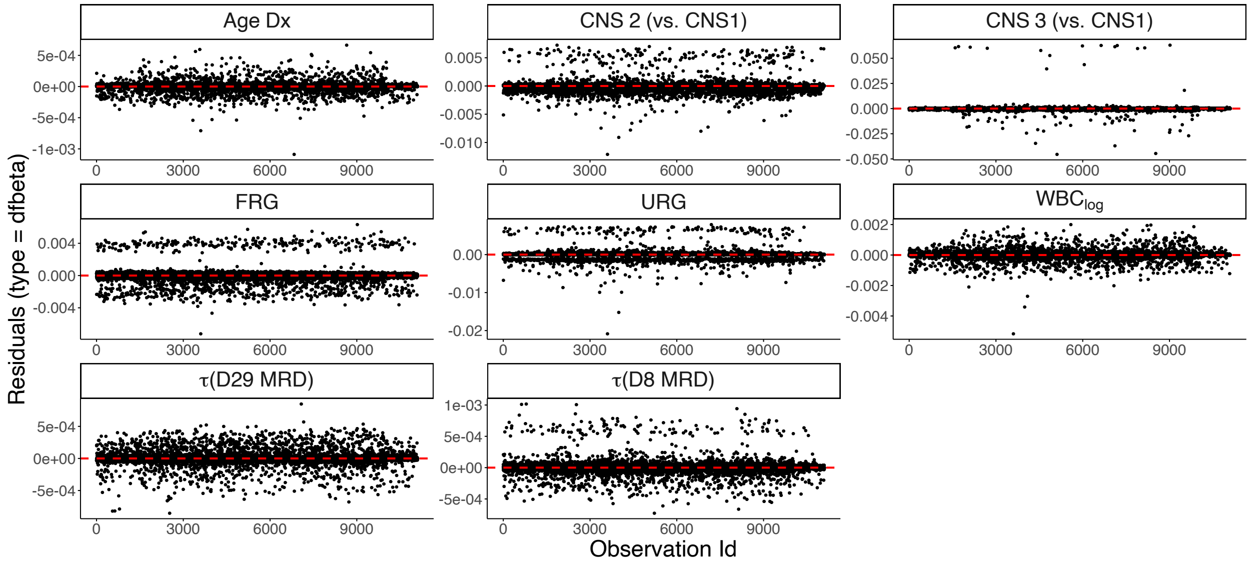

Supplemental Figure 4. Optimism-corrected calibration (B=200 bootstrap resamples) curves for the PI_{COG} model. A) Calibration curve in the training data. Blue solid line is predicted vs. observed survival probability at 5 years, while grey solid line through (0,1) is perfect calibration for reference. Distribution of predicted survival probabilities is shown across the top as a histogram. B) Calibration curves in the testing data stratified by study protocol (NCI high risk AALL0232 vs. NCI standard risk AALL0331). Blue solid line is predicted vs. observed survival probability at 5 years, while grey solid line through (0,1) is perfect calibration for reference. Distribution of predicted survival probabilities is shown across the top as a histogram. Mean |error|, mean absolute prediction error.

A)

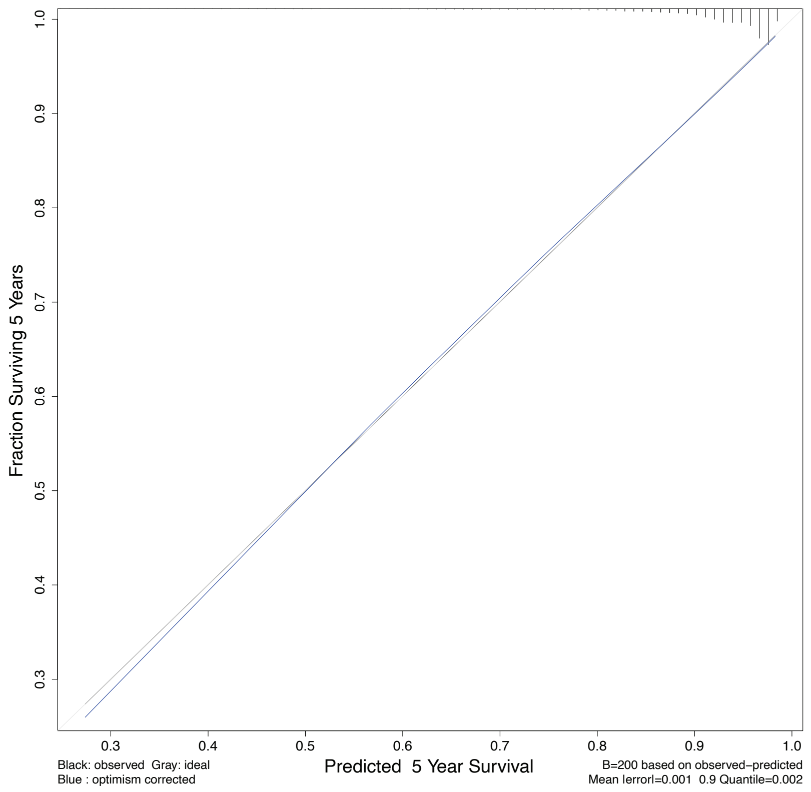

B)

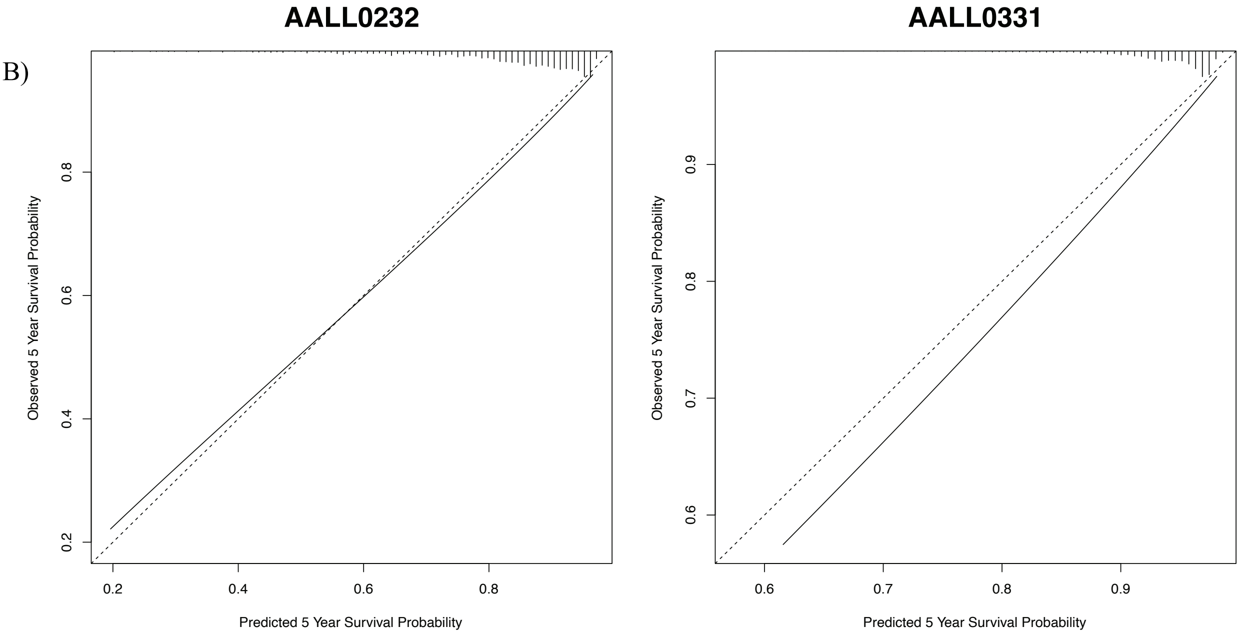

Supplemental Figure 5. Stratified Kaplan-Meier curves for relapse-free survival probability within each PI_{COG}-defined risk group and corresponding risk tables. A) Kaplan-Meier Curves in the training dataset. B) Kaplan-Meier curves in the testing dataset.

A)

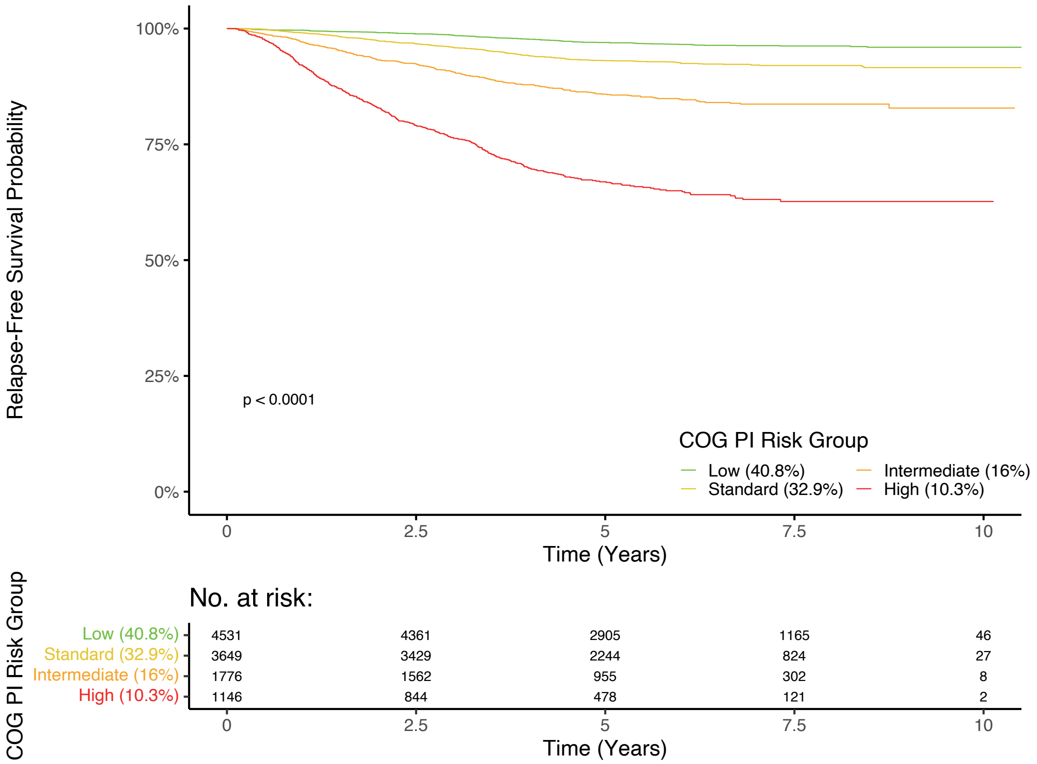

B)

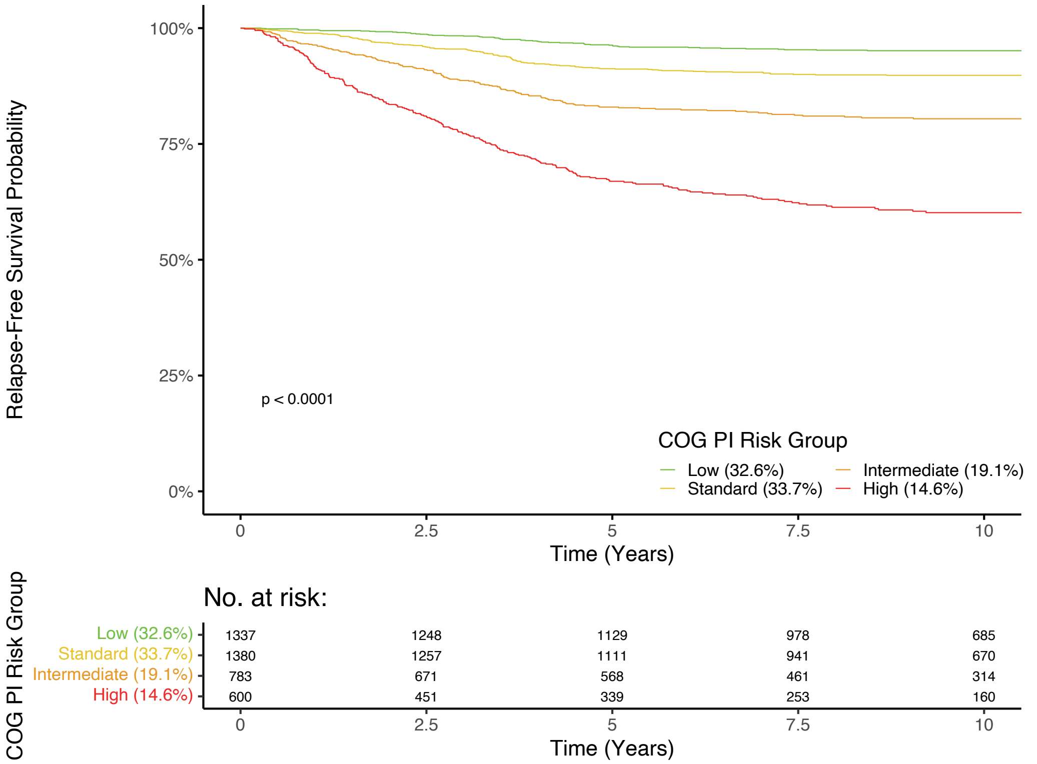

Supplemental Figure 6. Overlaid stratified Kaplan-Meier curves for relapse-free survival probability within each PI_{COG}-defined risk group. Solid lines indicate training dataset and dashed lines indicate testing dataset. Corresponding risk tables for training/testing stratified Kaplan-Meier curves are found in Supplemental Figures 5A and 5B, respectively.

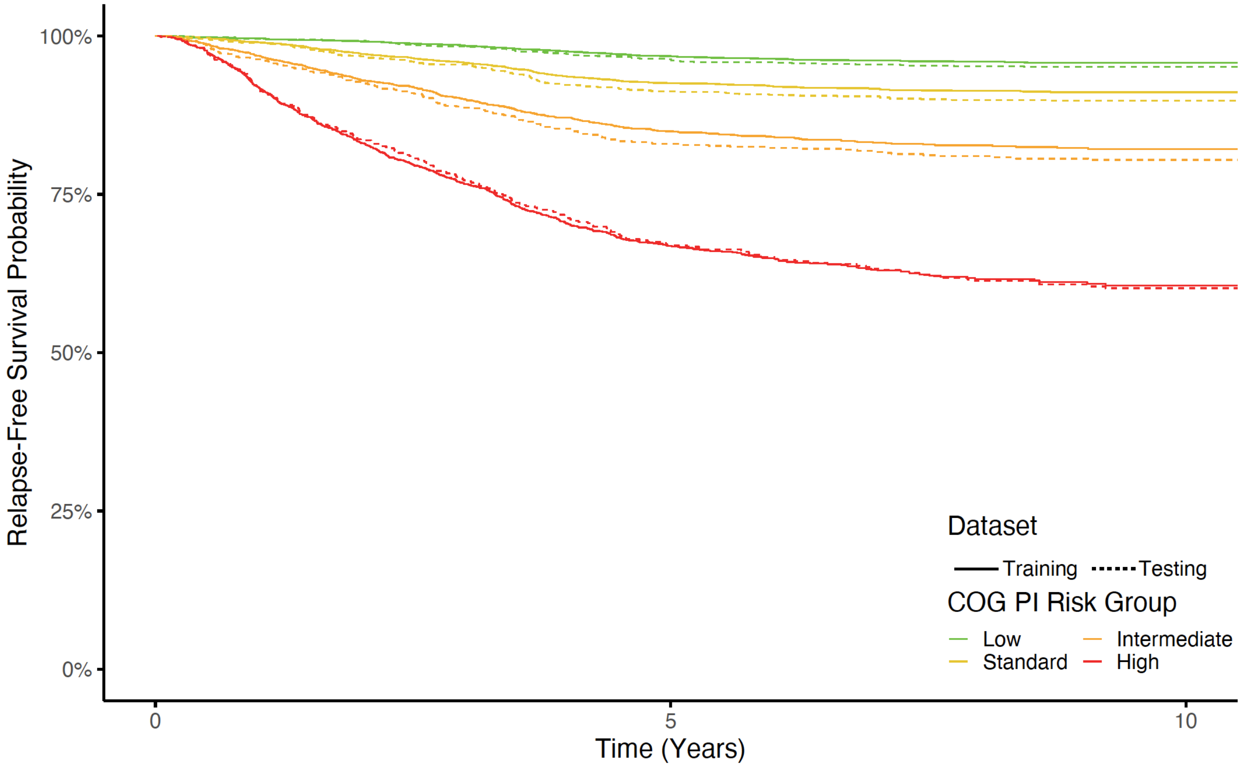

Supplemental Figure 7. Stratified density plots of the distribution of the PI_{COG} with CPE-defined risk groups indicated by text (Low, Standard, Intermediate, and High) and color. Risk group defining cutpoints of the PI_{COG} that maximize the CPE are marked by dashed vertical lines. A) Density plot in the training dataset. B) Density plot in the testing dataset.

A)

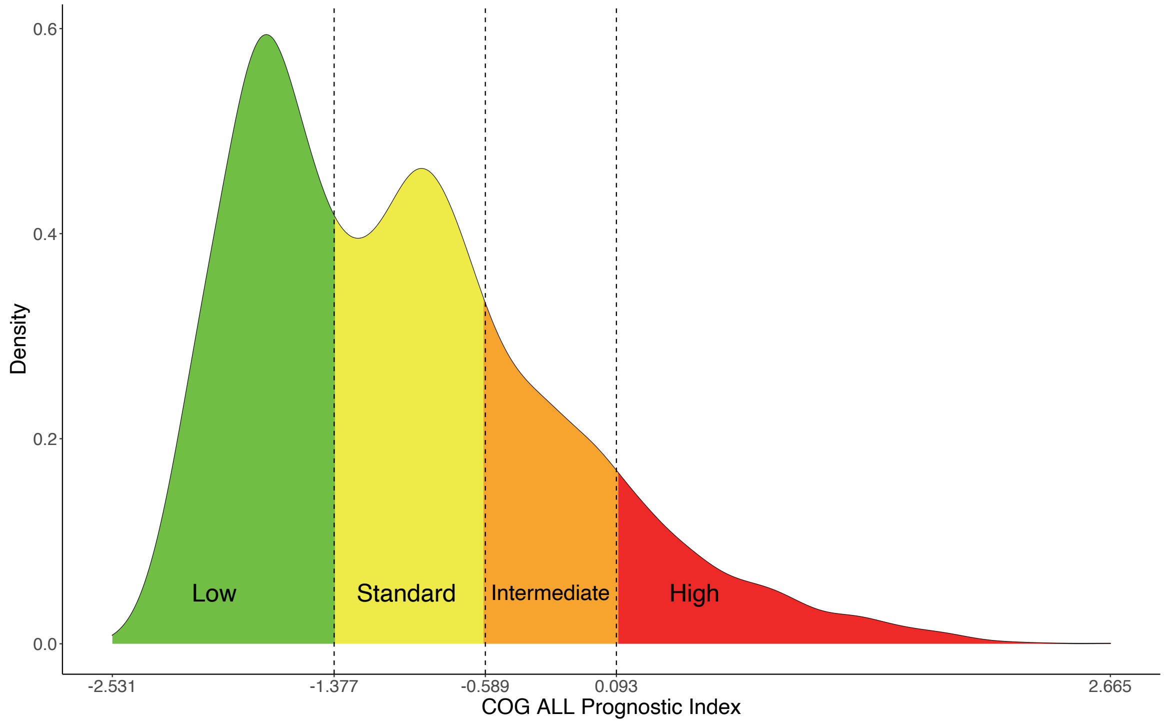

B)

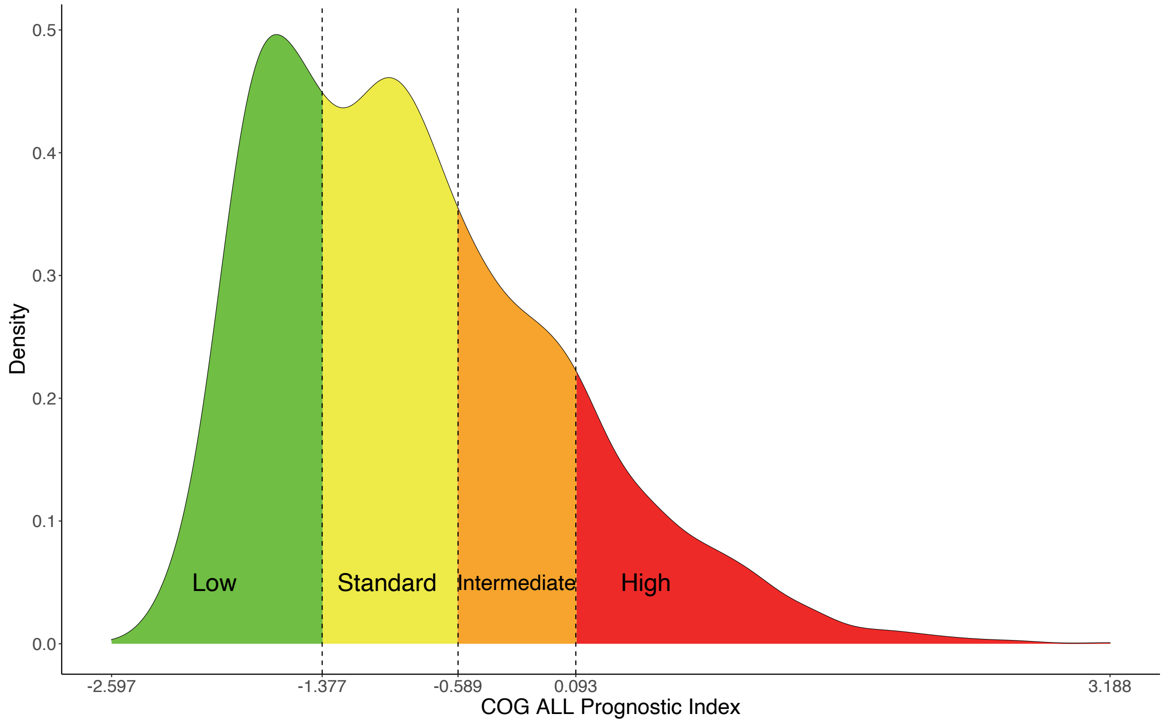

Supplemental Figure 8. Forest plots of the overall calibration slope (log-Hazard ratio) in the testing data. Point estimates of the log-transformed hazard ratios associated with the PI_{COG} within each variable category are represented on the forest plot as blue squares (size of square scaled by within-category sample size) with 95% confidence intervals. Perfect calibration is represented on the forest plot with a solid grey line at log(HR)=1. Log(HR), log-Hazard ratio; n, total number of patients in variable category; n. Events, total number of relapses in variable category; C-Index, concordance index.

Variable	Category	log(HR)	n	n. Events	C−Index
Gender	Female	0.95	1929	226	0.737
	Male	0.91	2171	322	0.737
Race/Ethnicity	Hispanic	0.94	999	179	0.736
	Non−Hispanic White	0.97	2328	259	0.738
	Non−Hispanic Black	0.82	253	48	0.735
	Non−Hispanic Asian	0.88	172	13	0.737
	Non−Hispanic Other	0.77	35	4	0.766

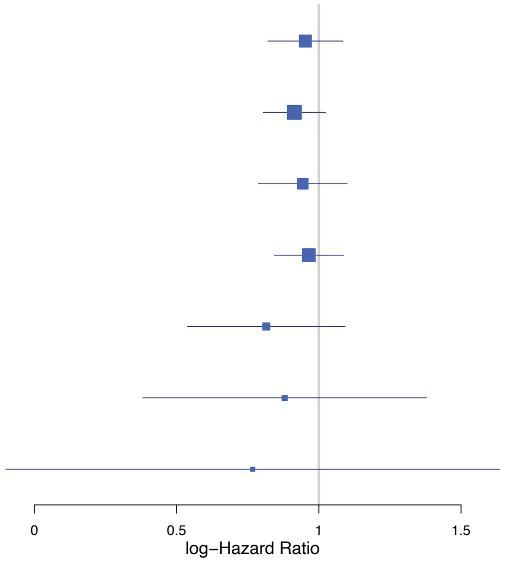

References

1. Tasian SK, Loh ML, Hunger SP. Philadelphia chromosome-like acute lymphoblastic leukemia. *Blood*. 2017;130(19):2064-2072. Doi:10.1182/blood-2017-06-743252
2. Maese L, Tasian SK, Raetz EA. How is the Ph-like signature being incorporated into ALL therapy?. *Best Pract Res Clin Haematol*. 2017;30(3):222-228. doi:10.1016/j.beha.2017.06.001
3. Barrio I, Rodríguez-Alvarez MX, Meira-Machado L, Esteban C, Arostegui I. Comparison of two discrimination indexes in the categorisation of continuous predictors in time-to-event studies. *Sort*. 2017;41(1):73-92. doi:10.2436/20.8080.02.51
4. Barrio I and Rodríguez-Alvarez MX (2022). CatPredi: Optimal Categorisation of Continuous Variables in Prediction Models. R package version 1.3, https://CRAN.R-project.org/package=CatPredi
5. Royston P, Altman DG. External validation of a Cox prognostic model: Principles and methods. *BMC Med Res Methodol*. 2013;13(1). doi:10.1186/1471-2288-13-33
6. Fouodo CJK. (2018). survivalsvm: Survival Support Vector Analysis. R package version 0.0.5, https://CRAN.R-project.org/package=survivalsvm
7. Ishwaran H and Kogalur UB (2022). Fast Unified Random Forests for Survival, Regression, and Classification (RF-SRC), R package version 3.1.0.
8. Hothorn T, Buehlmann P, Kneib T, Schmid M, and Hofner B (2022). mboost: Model-Based Boosting, R package version 2.9-7, https://CRAN.R-project.org/package=mboost.
9. Bischl B, Lang M, Kotthoff L, Schiffner J, Richter J, Studerus E, Casalicchio G, Jones Z (2016). "mlr: Machine Learning in R." Journal of Machine Learning Research, 17(170), 1-5. <https://jmlr.org/papers/v17/15-066.html>.
10. Fouodo CJK, König IR, Weihs C, Ziegler A, Wright MN. Support vector machines for survival analysis with R. *R J*. 2018;10(1):412-423. doi:10.32614/rj-2018-005
11. McLernon DJ, Giardiello D, Van Calster B, et al. Assessing Performance and Clinical Usefulness in Prediction Models With Survival Outcomes: Practical Guidance for Cox Proportional Hazards Models. *Ann Intern Med*. 2023;176(1):105-114. doi:10.7326/M22-0844
12. Harrell FE. *Regression Modeling Strategies: With Applications to Linear Models, Logistic and Ordinal Regression, and Survival Analysis (Second Edition)*; 2015.
